# Supplementary material for: Political development predicts reduced human cost of flooding
Source: Nat Commun. 2025 Nov 26;16:10128. doi: 10.1038/s41467-025-65914-6 (PMC12657876; doi:10.1038/s41467-025-65914-6)
Supplement: Supplementary file 1 — Supplementary Information [file 41467_2025_65914_MOESM1_ESM.pdf]

Supplementary Information for

Political development predicts reduced human cost of flooding.

Nature Communications (2025) doi: 10.1038/s41467-025-65914-6.

Paola Vesco* *et al.*

*Corresponding author. E-mail: paoves@prio.org

This PDF file includes:
Supplementary Text
Supplementary Figs. S1 to S40
Supplementary Tables S1 to S7

Contents

1 Summary statistics **3**

2 Sensitivity tests and alternative estimation strategies **15**
2.1 Aggregate measures of political development 15
2.2 Excluding socioeconomic indicators 18
2.2.1 Interactive effects of political development and GDP 20
2.2.2 Comparing the contribution of conflict and socioeconomic indicators 22
2.3 Excluding local indicators 23
2.4 Omitting continent-level random effects 25
2.5 Omitting yearly trends 27
2.6 Flood exposure at least 1,000 people 29
2.7 Deadly floods 31
2.8 Alternative training/test split 33
2.9 Random training/test split 35
2.10 Excluding influential data points 37
2.10.1 Excluding the most severe flood event 37
2.10.2 Excluding Myanmar 40
2.10.3 Excluding India 42
2.10.4 Excluding China 44
2.10.5 Excluding Bangladesh 46
2.11 Alternative estimation strategy: random forest models 48
2.12 Alternative estimation strategy: fixed effects models 49

3 Counterfactual analysis **52**

1 Summary statistics

This section provides descriptive statistics and plots for the main dataset, used to generate the results documented in Vesco, P., Von Uexkull, N., Vestby, J. & Buhaug, H., Political development predicts reduced human cost of flooding, *Nature Communications* (2025) doi: 10.1038/s41467-025-65914-6.

Accountability and government effectiveness are proxied by the "e_wbgi_vae" and "e_wbgi_gee" World Governance Indicators respectively [1], theoretically ranging from -2.5 to 2.5.

Inclusion and rule of law are measured respectively by the "v2xpe_exlsocgr" and "v2x_rule" index from V-dem [1], ranging from 0 to 1. In the original "v2xpe_exlsocgr" indicator, lower values reflect lower levels of exclusion (i.e., more inclusive outcomes). To ensure that higher values represent higher inclusion, we invert the original scale by subtracting the original value to 1.

Conflict history is a country-level decay function of the number of per-capita battle-related deaths from organized political violence, with a 2-years half-life (logged); local conflict is measured as the (logged) number of battle-related deaths in the flooded area. Both conflict variables are drawn from the geo-referenced version of the Uppsala Conflict Data Program [2]. All variables are lagged by 1 year.

Local development is computed as the average of three sub-national components – life expectancy, income, and years of schooling – derived from the local HDI dataset [3]. Each individual component is weighted by the extent of the area exposed to the flood before averaging.

As follows, we provide separate statistics for the training (2000–14) and test (2015–18) samples. These statistics reveal considerable differences in the sample characteristics for some variables, reflecting the fact that the samples are made up of distinct sets of flood events occurring in a varying set of countries, and are sensitive to spatio-temporal variation. This represents an important challenge to the prediction.

Table S1. Descriptive statistics for the training set (2000–14).

Variable	Missing	Mean	SD	Median	Min	Max
Flood mortality	0	83.95	1862.03	0.50	0.00	79828.00
Flood severity	0	1.30	0.41	1.00	1.00	2.00
Flood duration	0	2.80	1.01	2.83	0.00	6.04
Past flood events	0	2.75	1.40	2.77	0.00	5.24
Exposed population	0	8.37	2.83	8.25	0.54	16.27
Rough terrain	0	1.37	1.06	1.05	0.02	6.74
Tropical flood dummy	0	0.08	0.28	0.00	0.00	1.00
Local HDI	0	3.39	0.22	3.37	2.87	4.00
National GDP pc	0	7.92	1.53	7.72	4.71	11.18
Accountability	0	-0.15	0.96	-0.14	-2.23	1.69
Inclusion	0	0.61	0.24	0.58	0.09	0.98
Gov. effectiveness	0	-0.05	0.88	-0.16	-1.74	2.37
Rule of law	0	0.53	0.29	0.52	0.04	1.00
Conflict history	0	0.81	1.16	0.19	0.00	6.61
Local conflict	0	1.16	2.04	0.00	0.00	9.05
Electoral Democracy	0	0.53	0.27	0.54	0.02	0.91

The dataset contains 1,914 flood-country observations in 140 countries.

Note: All independent variables are lagged by 1 year (12 months) and all non-index independent variables are logged. Missing values for the independent variables were filled in by linear interpolation.

Table S2. Descriptive statistics for the test set (2015–18).

Variable	Missing	Mean	SD	Median	Min	Max
Flood mortality	0	8.33	27.58	0.00	0.00	244.00
Flood severity	0	1.42	0.35	1.50	1.00	2.00
Flood duration	0	2.53	0.82	2.48	0.69	4.34
Past flood events	0	2.80	1.24	2.71	0.00	4.81
Exposed population	0	8.16	2.64	8.07	2.26	15.16
Rough terrain	0	1.31	1.04	1.01	0.02	5.30
Tropical flood dummy	0	0.08	0.27	0.00	0.00	1.00
Local HDI	0	3.47	0.19	3.46	3.01	3.97
National GDP pc	0	8.55	1.28	8.65	5.50	11.04
Accountability	0	-0.10	0.83	-0.08	-1.91	1.54
Inclusion	0	0.63	0.23	0.64	0.11	0.98
Gov. effectiveness	0	-0.03	0.78	-0.05	-2.14	2.23
Rule of law	0	0.53	0.27	0.55	0.01	1.00
Conflict history	0	0.52	0.97	0.03	0.00	6.28
Local conflict	0	1.02	1.98	0.00	0.00	8.14
Electoral Democracy	0	0.53	0.25	0.54	0.08	0.92

The dataset contains 311 flood-country observations in 73 countries.
Note: All variables are lagged by 12 months and all non-index variables are logged. Missing values for the independent variables were filled in by linear interpolation.

Table S3. Between and within country standard deviation of political indicators for the full sample (2000–18, N=2225).

Variable	Within-country SD	Between-country SD	Ratio (Within/Between)
Accountability	0.94	0.13	0.13
Inclusion	0.26	0.02	0.06
Gov. effectiveness	0.95	0.13	0.14
Rule of law	0.30	0.03	0.10
Conflict history	44.97	12.67	0.28
Local conflict	407.67	116.76	0.29

a)

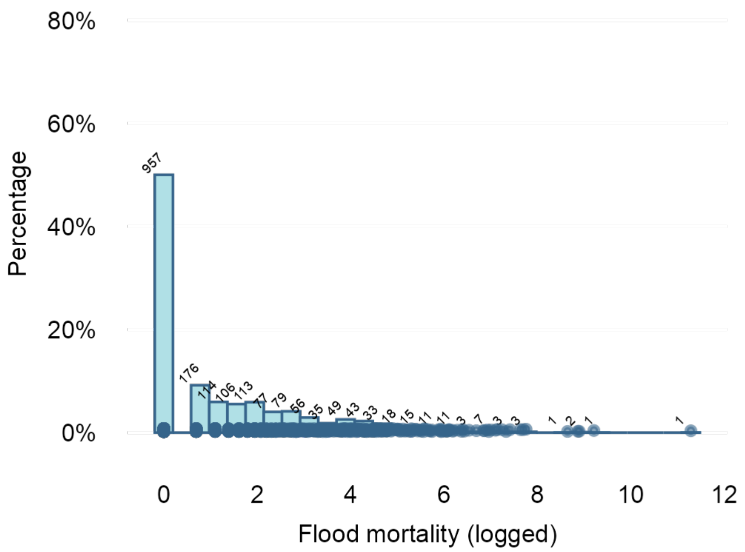

b)

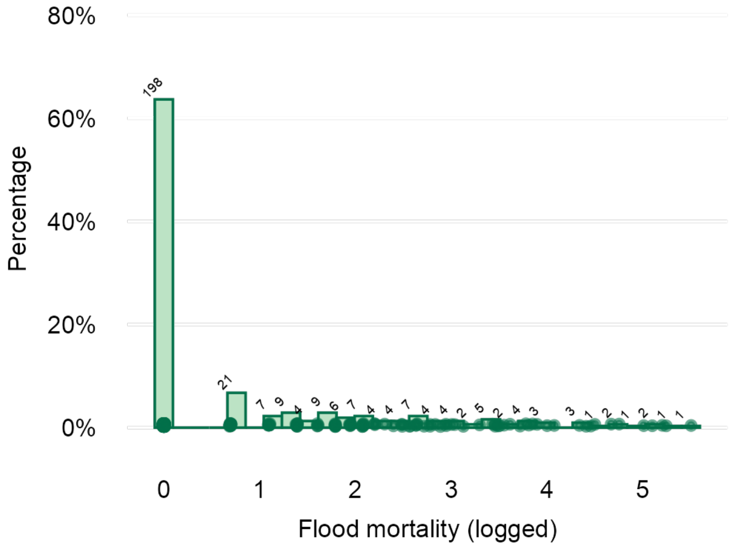

Fig. S1. Histograms of flood mortality for the training set (a) 2000–14, N = 1,914 (blue) and the test set (b) 2015–18, N=311 (green).

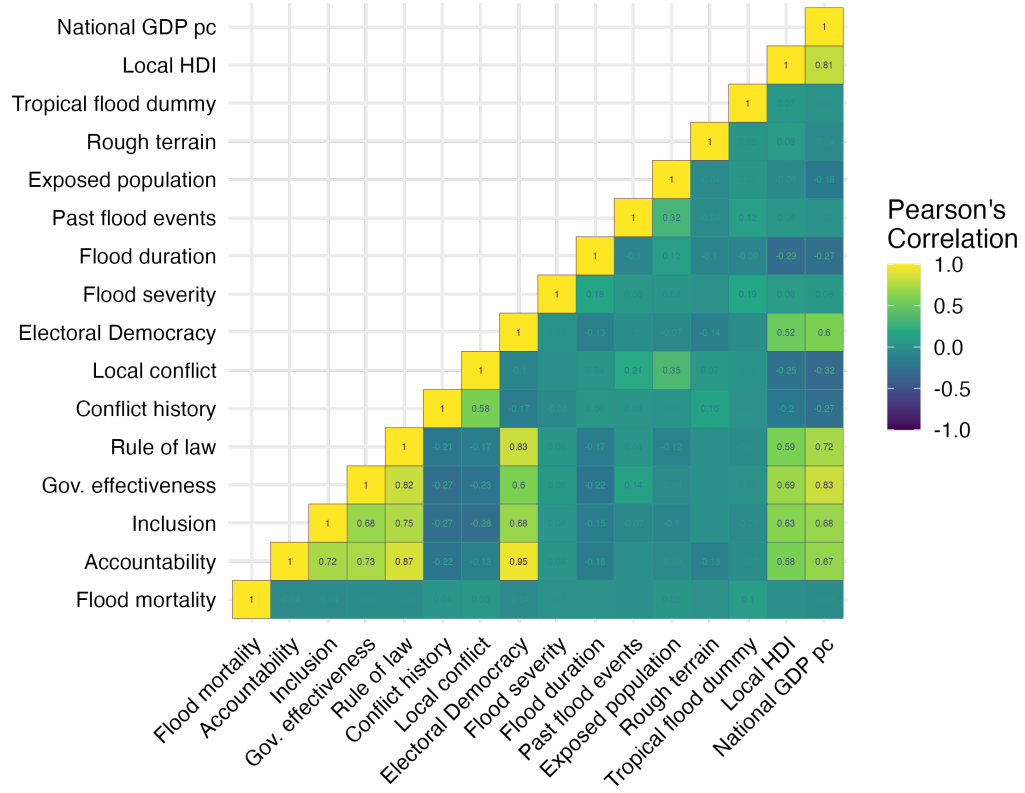

Fig. S2. Correlation matrix (2000–18, N=2,225). Pairwise Pearson's correlation between all variables in the dataset. Color range denotes the pairwise correlation value, from -1 (blue) to 1 (yellow).

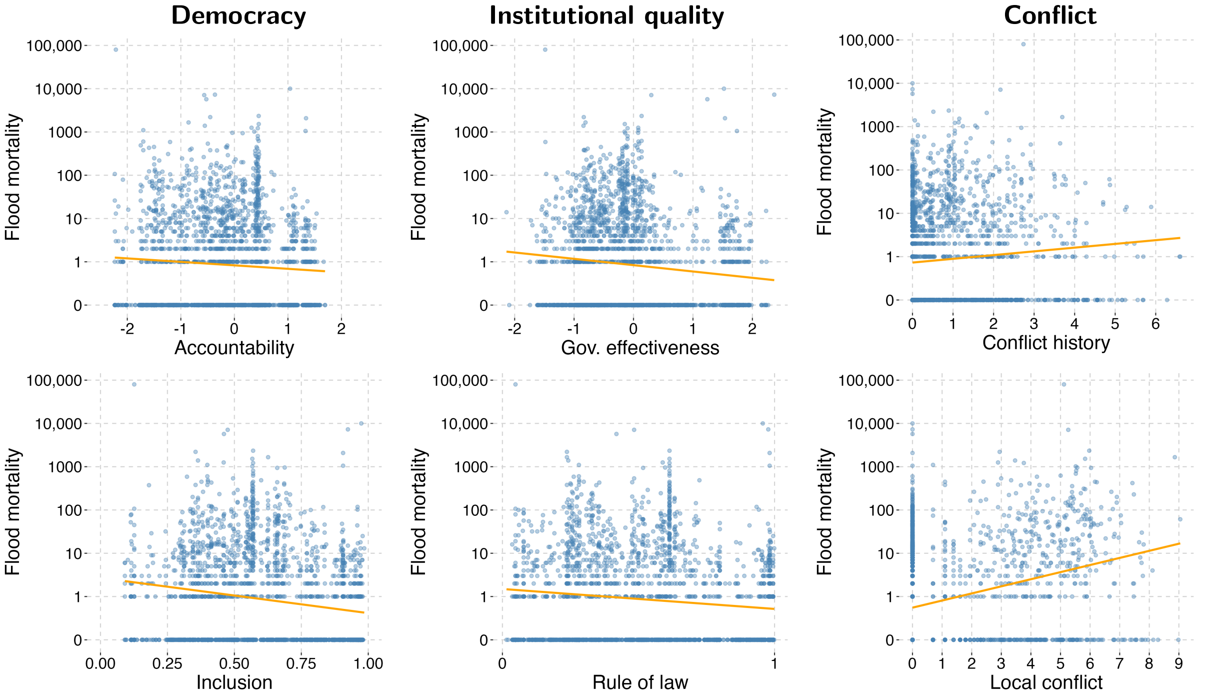

Fig. S3. Individual correlation plots between flood mortality and political development (2000–18, N=2,225). Orange lines denote linear trends.

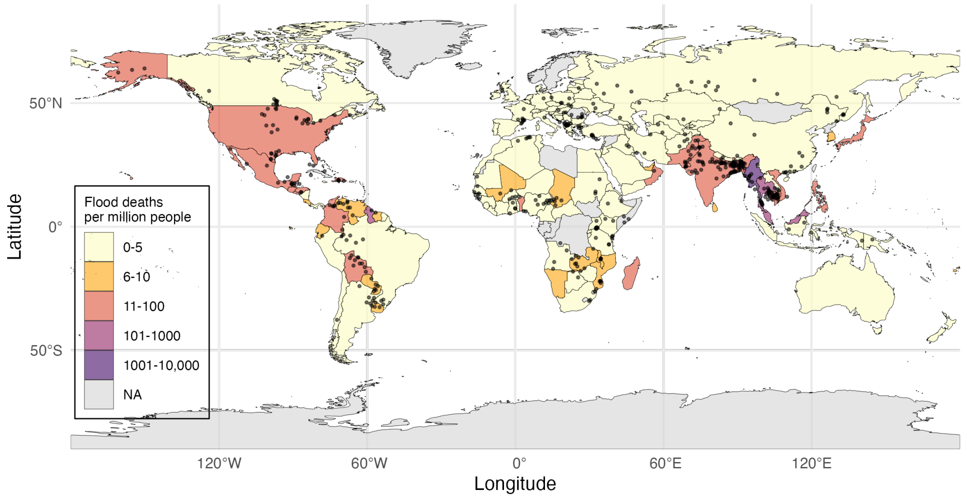

a) Training set (2000–14)

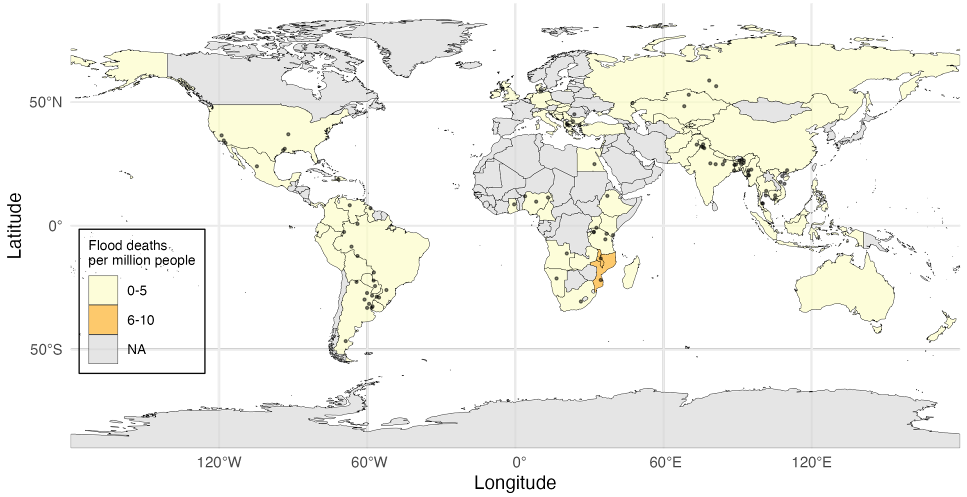

b) Test set (2015–18)

Fig. S4. Map of flood location and impact by country, for (a) the training set (N=1,914) and (b) the test set (N=311). The dots denote the centroids of the flooded areas of all events included in the analysis. Country colors reflect a simple categorization of total flood deaths per million people in the period, from yellow (low impact) to purple (high impact). Gray territories have missing information (i.e., there are no recorded flood events in the Global Flood Database, the flood polygons do not overlap with the geo-referenced human settlement data, or the country is not represented in the training/test sample as it did not experience any flood during that period).

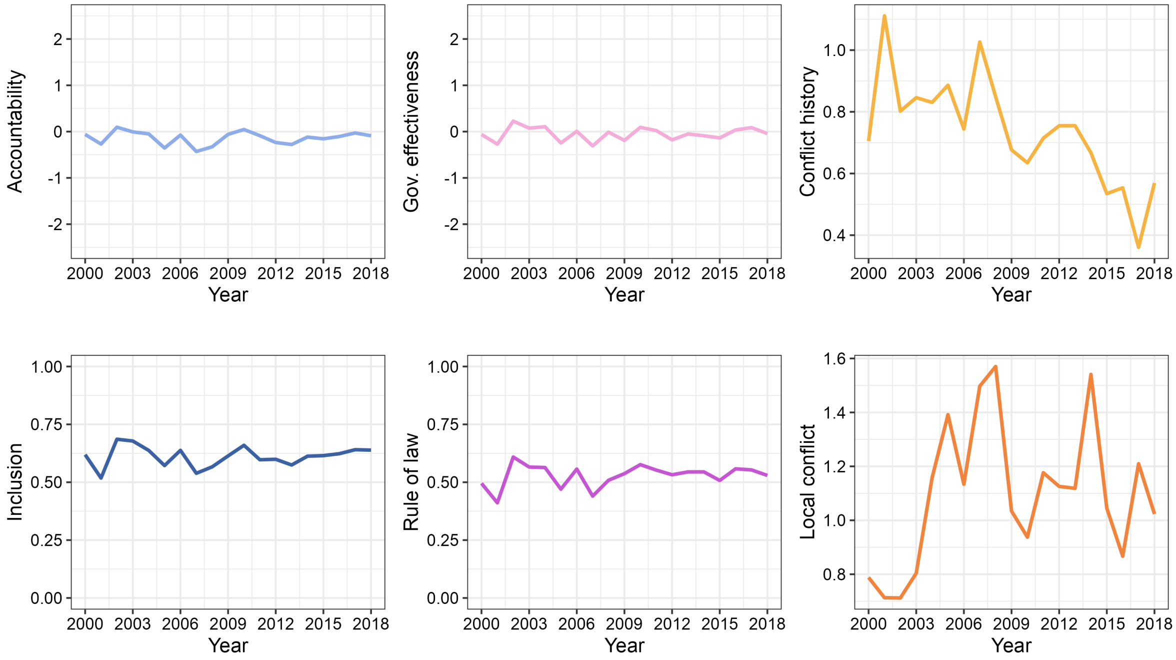

Fig. S5. Temporal trends (yearly) of all political development indicators aggregated across all countries in the full dataset (2000–2018, N=2,225). The y-axis is scaled to represent the full range of each index variable. Color shades represent political development dimensions: blue for democracy, pink for gov. effectiveness, orange for peace breakdown.

Additional details for the main specification

	Models						
	**Baseline**	**Accountability**	**Gov. effectiveness**	**Inclusion**	**Rule of law**	**Conflict history**	**Local conflict**
Exposed population	1.627<br>[1.558, 1.701]	1.648<br>[1.575, 1.725]	1.631<br>[1.561, 1.706]	1.632<br>[1.559, 1.707]	1.639<br>[1.567, 1.715]	1.633<br>[1.562, 1.708]	1.596<br>[1.526, 1.669]
Flood severity	4.689<br>[3.479, 6.371]	4.745<br>[3.524, 6.454]	4.761<br>[3.506, 6.480]	4.734<br>[3.511, 6.448]	4.699<br>[3.486, 6.375]	4.460<br>[3.308, 6.021]	4.266<br>[3.149, 5.787]
Flood duration	0.615<br>[0.543, 0.697]	0.594<br>[0.522, 0.675]	0.612<br>[0.539, 0.695]	0.612<br>[0.539, 0.695]	0.604<br>[0.530, 0.686]	0.615<br>[0.542, 0.697]	0.622<br>[0.549, 0.703]
Past flood events	1.334<br>[0.863, 1.964]	1.324<br>[0.900, 1.847]	1.334<br>[0.900, 1.906]	1.335<br>[0.888, 1.967]	1.327<br>[0.920, 1.848]	1.253<br>[0.732, 1.975]	1.266<br>[0.800, 1.900]
Rugged terrain	0.899<br>[0.779, 1.040]	0.849<br>[0.732, 0.987]	0.887<br>[0.767, 1.027]	0.892<br>[0.772, 1.033]	0.874<br>[0.754, 1.012]	0.843<br>[0.732, 0.973]	0.879<br>[0.763, 1.014]
Tropical flood	31.859<br>[21.184, 49.037]	27.326<br>[17.936, 42.360]	32.213<br>[21.332, 49.648]	30.660<br>[20.012, 48.023]	30.641<br>[20.441, 47.238]	28.778<br>[19.206, 43.993]	27.583<br>[18.241, 42.492]
National GDP per capita	1.099<br>[0.963, 1.258]	1.173<br>[1.018, 1.356]	1.168<br>[0.961, 1.424]	1.119<br>[0.965, 1.300]	1.183<br>[1.013, 1.384]	1.181<br>[1.033, 1.352]	1.199<br>[1.047, 1.374]
Local HDI	0.035<br>[0.011, 0.112]	0.038<br>[0.012, 0.125]	0.035<br>[0.011, 0.113]	0.035<br>[0.011, 0.115]	0.036<br>[0.011, 0.117]	0.050<br>[0.016, 0.162]	0.052<br>[0.016, 0.169]
Accountability		0.801<br>[0.668, 0.957]					
Gov. effectiveness			0.896<br>[0.684, 1.173]				
Inclusion				0.822<br>[0.403, 1.662]			
Rule of law					0.569<br>[0.296, 1.070]		
Conflict history						1.266<br>[1.133, 1.420]	
Local conflict							1.158<br>[1.092, 1.230]

Table S4. Negative binomial models with continent-level random effects, as in the main specification (in-sample, N=1,914). Incidence rate ratios are displayed for all coefficients from the random effects models in the main specification. 95% credible intervals are displayed in brackets.

a) Convergence

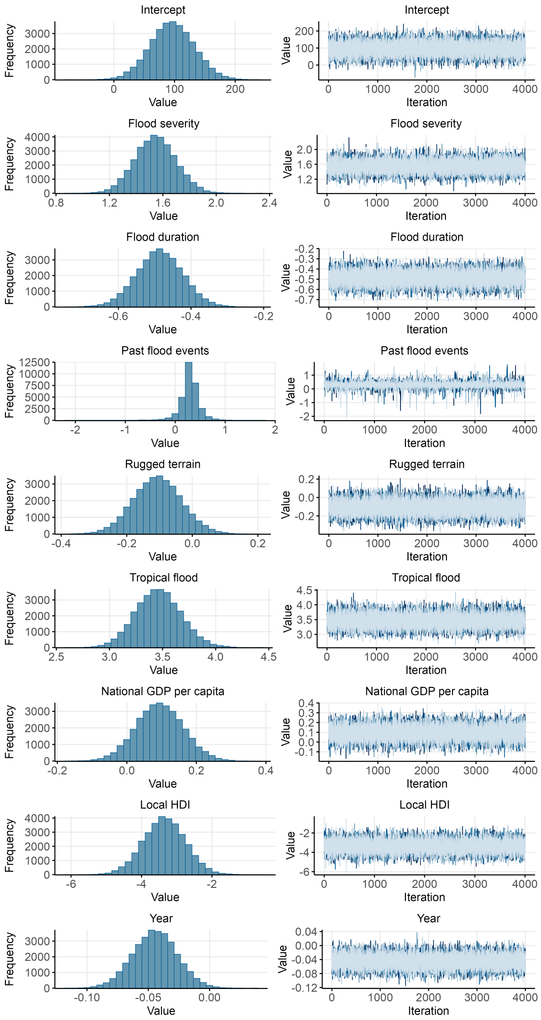

b) Predictor statistics

Predictor	R-hat	Bulk ESS	Tail ESS
Intercept	1	36429	25240
Flood severity	1	37959	24492
Flood duration	1	33890	24914
Past flood events	1	9489	6564
Exposed population	1	35727	24322
Rugged terrain	1	38252	24792
Local HDI	1	24443	24494
National GDP per capita	1	28370	24011
Tropical flood	1	40198	20082
Year trend	1	37381	24865

c) K-statistic evaluation

K-statistic	Count	%	Evaluation
$(-Inf, 0.7]$	1910	99.8%	Good
$(0.7, 1]$	1	0.1%	Poor
$(1, Inf)$	3	0.2%	Very poor

Fig. S6. In-sample regression diagnostics for the baseline model (N=1,914). (a) posterior distribution and trace plots; (b) convergence diagnostics for the baseline model predictors. All model parameters have an R-hat equal to 1 and high Bulk and tail Effective Sample Size (ESS) values, suggesting good model convergence and reliable estimates. (c) K-diagnostics assessing the reliability of the Pareto-smoothed importance sampling Leave-one-out Cross Validation (PSIS-LOO-CV). The k-statistics suggest that the PSIS-LOO-CV method works well for the vast majority of our data.

**Model**	**In-sample (2000–2014)**	**Out-of-sample (2015–2018)**
Local conflict	9827.31	1196.19
Conflict history	9832.68	1198.36
Accountability	9848.85	1201.99
Rule of law	9852.40	1205.41
Baseline	9853.17	1199.41
Electoral democracy	9854.80	1200.43
Gov. effectiveness	9856.11	1202.96
Inclusion	9856.87	1200.09

Table S5. Relative predictive performance of political development models on flood mortality, based on the Watanabe-Akaike information criteria (WAIC). The models are the same as in the main specification presented in the article. Models are ranked by best in-sample performance (N=1,914). WAIC is based on the computed log pointwise posterior predictive density and adds a correction for the number of parameters to adjust for overfitting. Unlike elpd, WAIC does not rely on cross-validation.

2 Sensitivity tests and alternative estimation strategies

To assess the robustness of the results reported in the main article, we conducted a series of tests using alternative model and sample specifications. On the following pages, we document conditional effect plots, elpd scores and stacking weights for models that: (i) use aggregate predictors of political development (Fig. S7–S8); (ii) exclude socioeconomic predictors to assess political development contributions without socioeconomic development (Fig. S9–S10); iii) assess the interactions between political and socioeconomic development (Fig. S11–S12); (iv) exclude local predictors (Fig. S14–S15); (v) omit the continent random effects (Fig. S16–S17) and yearly trends (Fig. S18–S19; (vi) are limited to floods with at least 1,000 people exposed (Fig. S20–S21); (vii) are limited to deadly floods (Fig. S22–S23); (viii) use an alternative specification of training and test samples (Fig. S24–S27); ix) exclude outliers and influential countries (Fig. S28 – Fig. S37). In addition, we document the results of x) a set of random forest models (Fig. S38), and xi) two-way fixed effects Poisson regression models controlling for both country and year effects (Table S6 and Fig. S39). The findings of these tests overall add confidence in the results discussed in the main article.

2.1 Aggregate measures of political development

In this section, we move from employing discrete indicators in our primary analysis to utilizing aggregate measures of political development. These additional tests aim to determine if broader indicators yield comparable patterns in how political development influences flood mortality. To this end, we estimate joint predictive performance of indicators belonging to the same dimension of political development in models which otherwise are identical to our main specification. We separately use a different, widely-used composite index of Electoral democracy as alternative measure for the democracy dimension. All models include the same baseline indicators as the main specification. Overall patterns are in line with main results.

a) b)

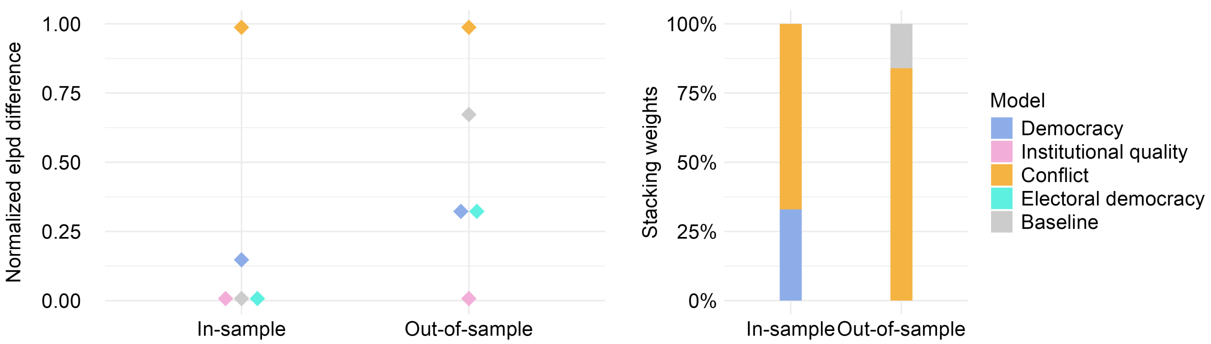

c)

	In-sample (2000-2014)			Out-of-sample (2015-2018)		
Model	elpd LOO	Δelpd	stacking weight	elpd LOO	Δelpd	stacking weight
Conflict	-4918 (117)	0 (0)	0.67	-594 (38)	0 (0)	0.84
Democracy	-4929 (118)	-12 (9)	0.33	-596 (38)	-2 (2)	0.00
Institutional quality	-4931 (118)	-14 (7)	0.00	-598 (38)	-3 (2)	0.00
Baseline	-4932 (119)	-14 (8)	0.00	-596 (38)	-1 (2)	0.16
Electoral democracy	-4932 (118)	-14 (8)	0.00	-596 (38)	-2 (2)	0.00

Fig. S7. Relative predictive performance of aggregate political development models on flood mortality. a) In-sample (2000–14, N=1,914) and out-of-sample (2015–18, N=311) expected log predictive density (elpd) for each model, obtained via leave-one-out cross-validation with Pareto-smoothed importance sampling, normalized such that the best-performing model scores 1 and the worst scores 0. **b)** In-sample and out-of-sample stacking weights for each model, reflecting the proportion of observations for which each model provides superior predictive performance. **c)** In-sample and out-of-sample elpd values (non-normalized) and stacking weights for each indicator model, ranked by in-sample elpd performance (values closer to zero indicate better fit; standard errors in parentheses). Each model includes all indicators of a given political development dimension (color) in addition to the baseline (grey). Colors reflect the political development dimension of the main predictor in the model (blue: democracy, turquoise: aggregate indicator of electoral democracy, pink: institutional quality, orange: peace breakdown). Note that we did not compute conditional effect plots for the aggregate models, as the political development indicators within each dimension are highly correlated (see Fig. S2).

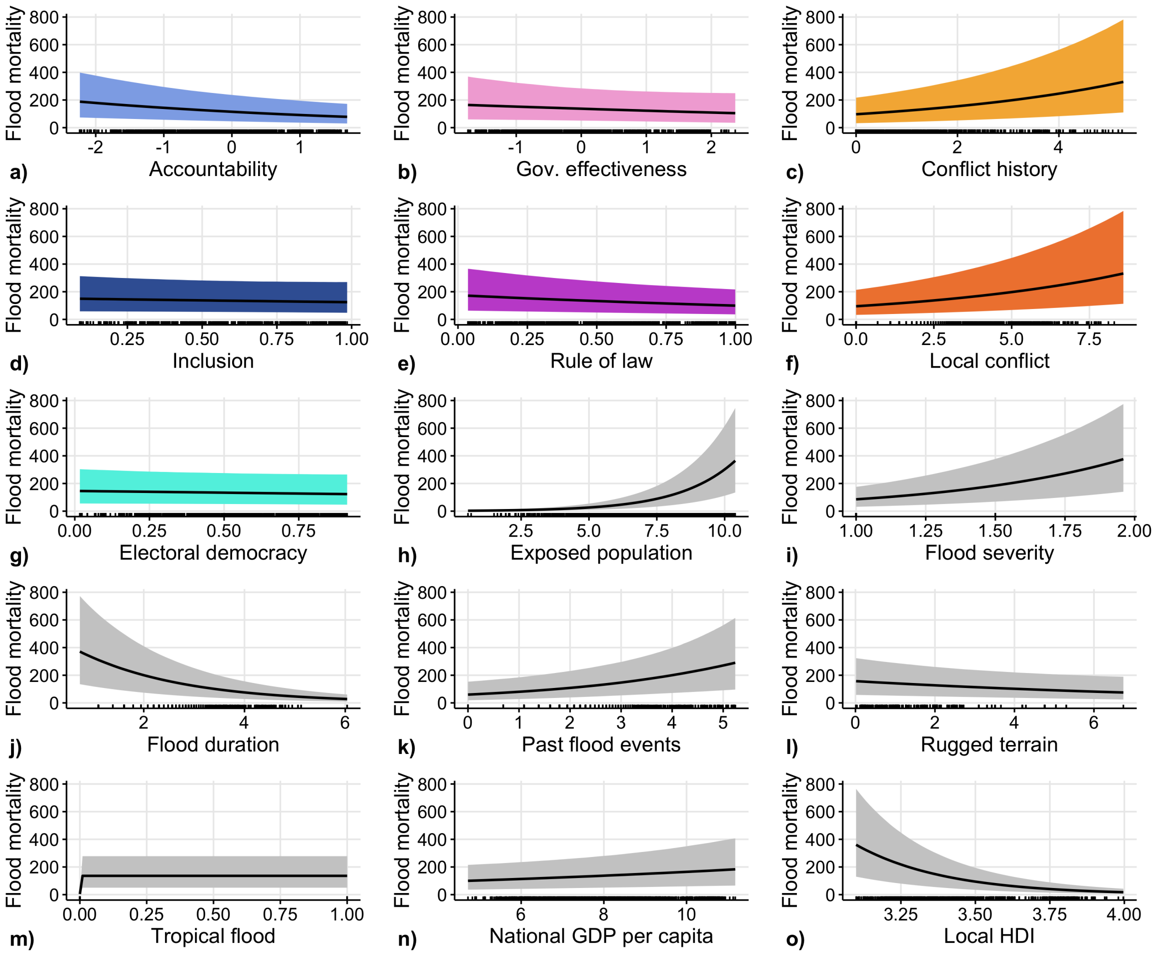

Fig. S8. Conditional effect plots for the aggregate Electoral democracy index. Each plot (a-o) shows the in-sample median of the posterior predictive distribution (black line) and the surrounding 80% predictive interval (shaded area) for the selected indicator, based on 8 × 4,000 Markov chain Monte Carlo draws per model. The Electoral democracy index (turquoise) is drawn from V-Dem (v2x_polyarchy) [4]. The conditional effects for the other political indicators are produced from the main models presented in the article. All models are specified as Bayesian random-effects negative binomial regressions. All effects are computed by including the selected indicator of political development only (color), in addition to the baseline predictors (grey), holding other variables at their observed mean values. Colors reflect the political development dimension of the main predictor in the model (blue: democracy, pink: institutional quality, orange: peace breakdown). Rug plots display the distribution of data points. The plots are shown for a subset of observations excluding the 20% most severe conflict history events, although the underlying models were estimated on the complete training sample, 2000–14 (N=1,914).

2.2 Excluding socioeconomic indicators

Next, we estimate the models with national GDP per capita and local HDI excluded from the baseline. These additional analyses assesses whether political development influences disaster mortality when excluding socioeconomic indicators which may be bad controls, as they are affected by political development. The estimated magnitude of the conditional effects are in line with the main specification.

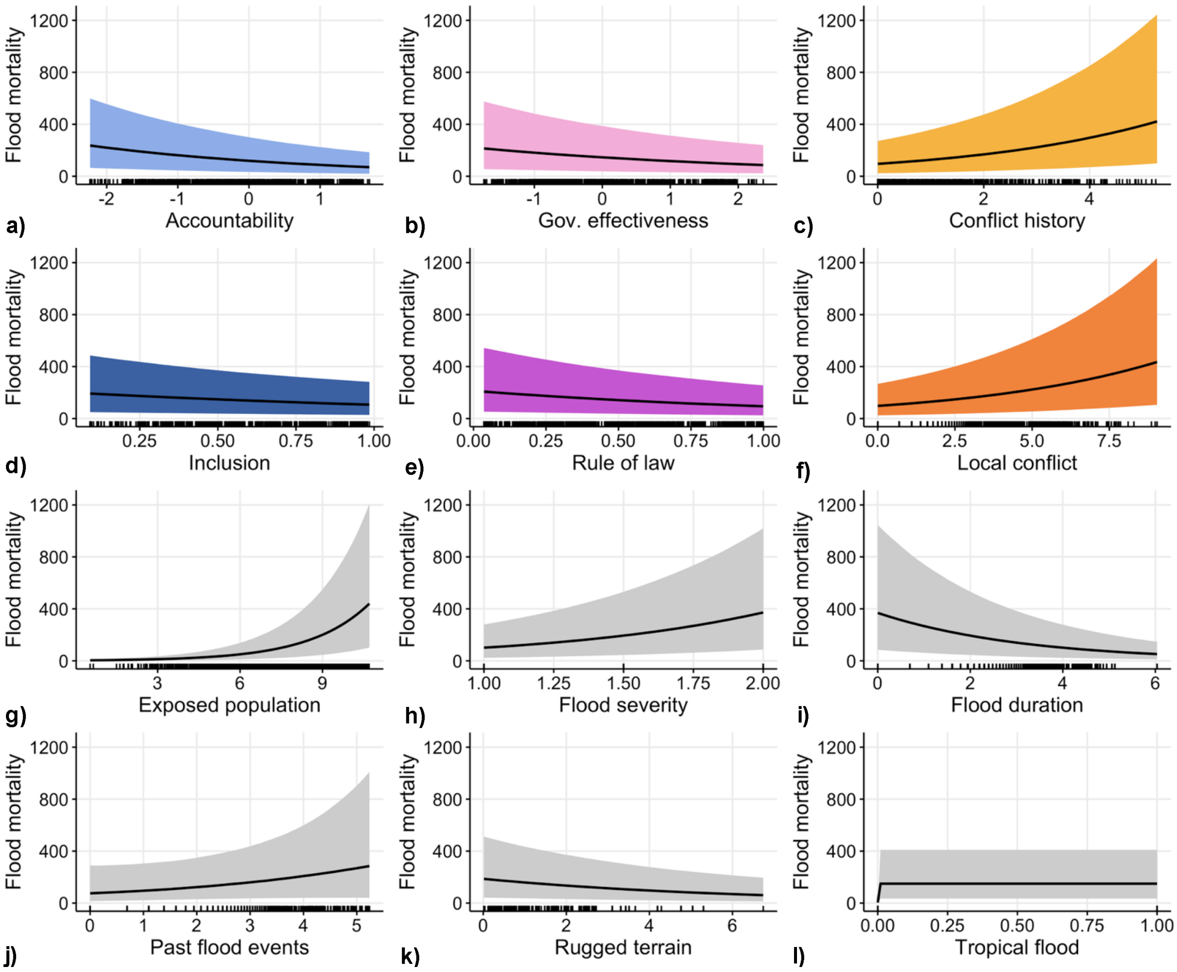

Fig. S9. Conditional effect plots for drivers of flood mortality, excluding socioeconomic indicators (GDP per capita and local HDI) from the baseline. Each plot (a-l) shows the in-sample median of the posterior predictive distribution (black line) and the surrounding 80% predictive interval (shaded area) for the selected indicator, based on 8 × 4,000 Markov chain Monte Carlo draws per model. All models are specified as Bayesian random-effects negative binomial regressions. All effects are computed by including the selected indicator of political development only (color), in addition to the baseline predictors (grey), holding other variables at their observed mean values. Colors reflect the political development dimension of the main predictor in the model (blue: democracy, pink: institutional quality, orange: peace breakdown). Rug plots display the distribution of data points. Plots are shown for a subset of observations excluding the 20% most severe conflict history events, although the underlying models were estimated on the complete training sample, 2000–14 (N=1,914).

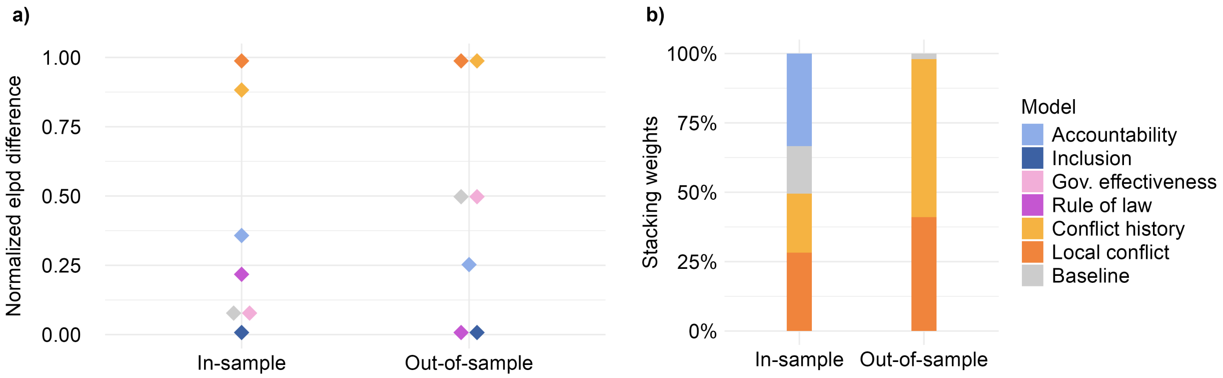

c)

	In-sample (2000-2014)			Out-of-sample (2015-2018)		
Model	elpd LOO	Δelpd	stacking weight	elpd LOO	Δelpd	stacking weight
Local conflict	-4921 (116)	0 (0)	0.28	-597 (37)	0 (1)	0.41
Conflict history	-4924 (115)	-3 (6)	0.21	-597 (37)	0 (0)	0.57
Accountability	-4937 (116)	-15 (12)	0.33	-600 (37)	-3 (1)	0.00
Rule of law	-4939 (116)	-18 (10)	0.00	-601 (37)	-4 (1)	0.00
Baseline	-4942 (117)	-21 (14)	0.17	-599 (37)	-2 (2)	0.02
Gov. effectiveness	-4942 (117)	-21 (10)	0.00	-599 (37)	-2 (2)	0.00
Inclusion	-4944 (117)	-23 (12)	0.00	-601 (37)	-4 (2)	0.00

Fig. S10. Relative predictive performance of political development models on flood mortality, excluding the socioeconomic indicators (GDP and local HDI) from the baseline. a) In-sample (2000–14, N=1,914) and out-of-sample (2015–18, N=311) expected log predictive density (elpd) for each model, obtained via leave-one-out cross-validation with Pareto-smoothed importance sampling, normalized such that the best-performing model scores 1 and the worst scores 0. **b)** In-sample and out-of-sample stacking weights for each model, reflecting the proportion of observations for which each model provides superior predictive performance. **c)** In-sample and out-of-sample elpd values (non-normalized) and stacking weights for each indicator model, ranked by in-sample elpd performance (values closer to zero indicate better fit; standard errors in parentheses). Each model includes all indicators of a given political development dimension (color) in addition to the baseline (grey). Colors reflect the political development dimension of the main predictor in the model (blue: democracy, pink: institutional quality, orange: peace breakdown).

2.2.1 Interactive effects of political development and GDP

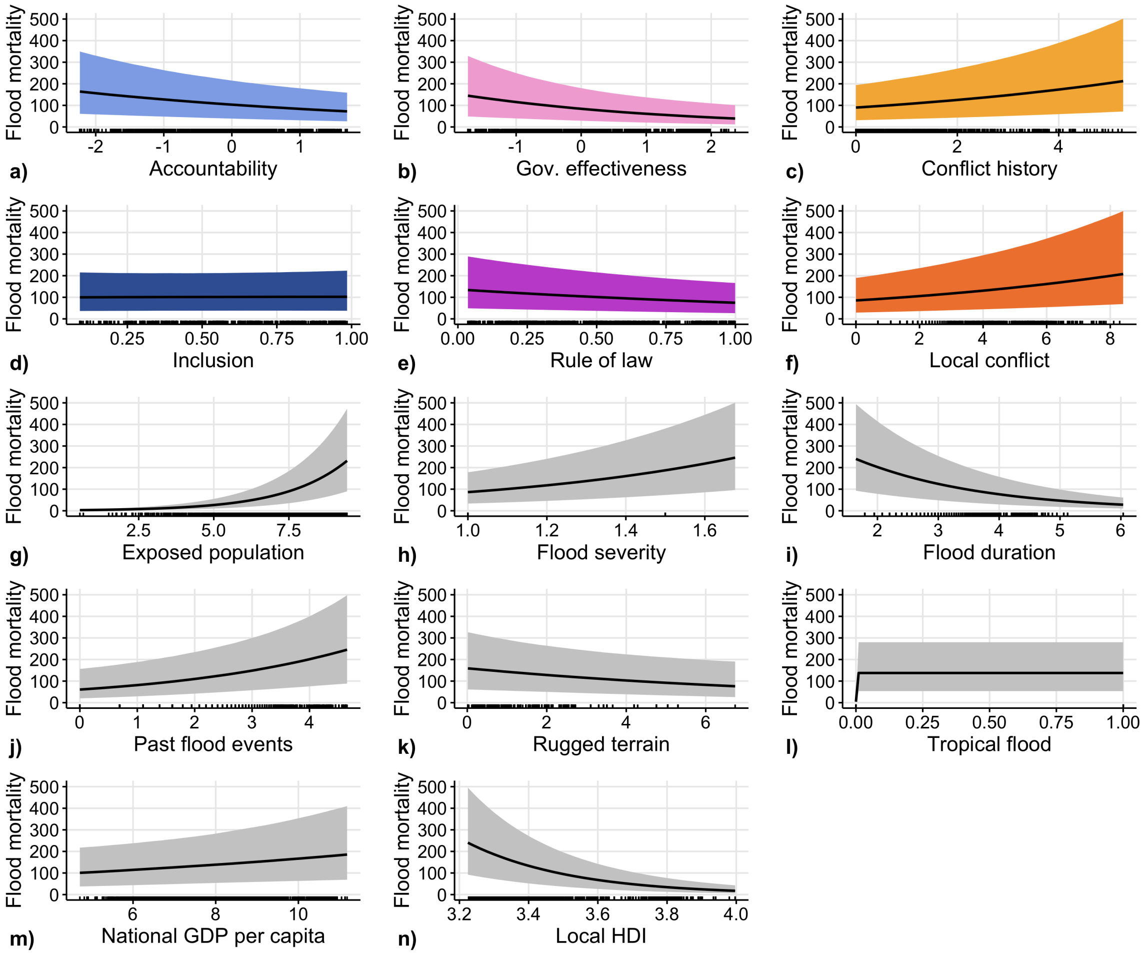

Fig. S11. Conditional effect plots for the models including interaction terms for political indicators and GDP. Each plot (a-n) shows the in-sample median of the posterior predictive distribution (black line) and the surrounding 80% predictive interval (shaded area) for the selected indicator, based on $8 \times 4{,}000$ Markov chain Monte Carlo draws per model. All models are specified as Bayesian random-effects negative binomial regressions. All effects are computed by including the selected indicator of political development only (color), in addition to the baseline predictors including GDP and Local HDI (grey), holding other variables at their observed mean values. The models include interactive terms of each political indicator with GDP per capita. Colors reflect the political development dimension of the main predictor in the model (blue: democracy, pink: institutional quality, orange: peace breakdown). Rug plots display the distribution of data points. Plots are shown for a subset of observations excluding the 20% most severe conflict history events, although the underlying models were estimated on the complete training sample, 2000–14 (N=1,914).

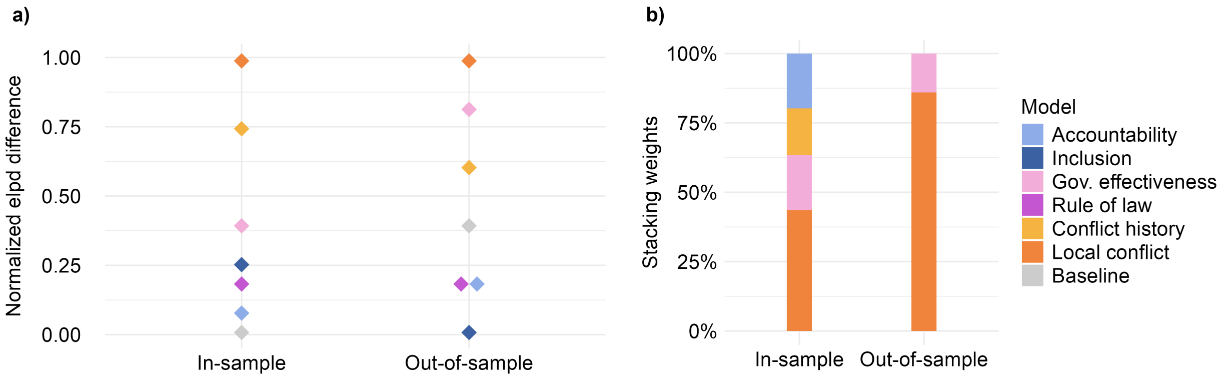

c)

	In-sample (2000-2014)			Out-of-sample (2015-2018)		
Model	elpd LOO	Δelpd	stacking weight	elpd LOO	Δelpd	stacking weight
Local conflict	-4916 (117)	0 (0)	0.44	-592 (38)	0 (0)	0.86
Conflict history	-4920 (117)	-4 (5)	0.17	-594 (38)	-2 (2)	0.00
Gov. effectiveness	-4926 (117)	-9 (8)	0.20	-593 (38)	-1 (2)	0.14
Inclusion	-4927 (117)	-11 (8)	0.00	-597 (38)	-5 (2)	0.00
Rule of law	-4928 (117)	-12 (7)	0.00	-596 (38)	-4 (1)	0.00
Accountability	-4930 (118)	-14 (8)	0.20	-596 (38)	-4 (2)	0.00
Baseline	-4931 (118)	-15 (9)	0.00	-596 (38)	-3 (2)	0.00

Fig. S12. Relative predictive performance of political development models on flood mortality including interaction terms for political indicators and GDP. a) In-sample (2000–14, N=1,914) and out-of-sample (2015–18, N=311) expected log predictive density (elpd) for each model, obtained via leave-one-out cross-validation with Pareto-smoothed importance sampling, normalized such that the best-performing model scores 1 and the worst scores 0. **b)** In-sample and out-of-sample stacking weights for each model, reflecting the proportion of observations for which each model provides superior predictive performance. **c)** In-sample and out-of-sample elpd values (non-normalized) and stacking weights for each indicator model, ranked by in-sample elpd performance (values closer to zero indicate better fit; standard errors in parentheses). Each model includes all indicators of a given political development dimension (color) in addition to the baseline, including GDP and Local HDI (grey). All models include interactive terms for each political indicator with GDP. Colors reflect the political development dimension of the main predictor in the model (blue: democracy, pink: institutional quality, orange: peace breakdown).

2.2.2 Comparing the contribution of conflict and socioeconomic indicators

We compare the predictive performance of the two conflict indicators against socioeconomic indicators by computing the ELPD and stacking weights for an ensemble of only two models: a baseline model (with national GDP and local HDI), and a conflict model that includes both conflict indicators in addition to the baseline features (with GDP and HDI). This comparison assesses the added predictive contribution of conflict beyond GDP per capita and local HDI, and reveals that the conflict model has by far the highest contribution to predictive performance both in-sample and out-of-sample.

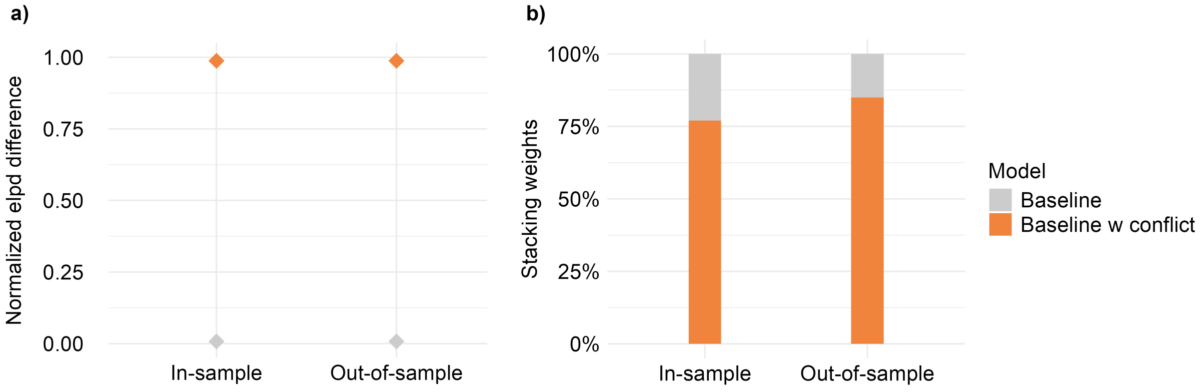

c)

	In-sample (2000-2014)			Out-of-sample (2015-2018)		
Model	elpd LOO	Δelpd	stacking weight	elpd LOO	Δelpd	stacking weight
Baseline w conflict	-4918 (117)	0 (0)	0.77	-594 (38)	0 (0)	0.85
Baseline	-4931 (118)	-13 (8)	0.23	-596 (38)	-1 (2)	0.15

Fig. S13. Relative predictive performance of conflict predictors of flood mortality vs socioeconomic predictors. The predictive performance compares a baseline model and a model with conflict history and local conflict added to the baseline features. **a)** In-sample (2000–14, N=1,914) and out-of-sample (2015–18, N=311) expected log predictive density (elpd) for each model, obtained via leave-one-out cross-validation with Pareto-smoothed importance sampling, normalized such that the best-performing model scores 1 and the worst scores 0. **b)** In-sample and out-of-sample stacking weights for each model, reflecting the proportion of observations for which each model provides superior predictive performance. **c)** In-sample and out-of-sample elpd values (non-normalized) and stacking weights for each indicator model, ranked by in-sample elpd performance (values closer to zero indicate better fit; standard errors in parentheses). The model includes all conflict indicators (orange) in addition to the baseline (grey). Colors reflect the political development dimension of the main predictor in the model (orange: peace breakdown).

2.3 Excluding local indicators

The main specification combines country-level indices with indicators measured specifically for the flooded areas (i.e. local conflict). To enable a more equal evaluation of indicator and model performance, we estimated the models without local conflict and local HDI. Results remain substantively unaltered.

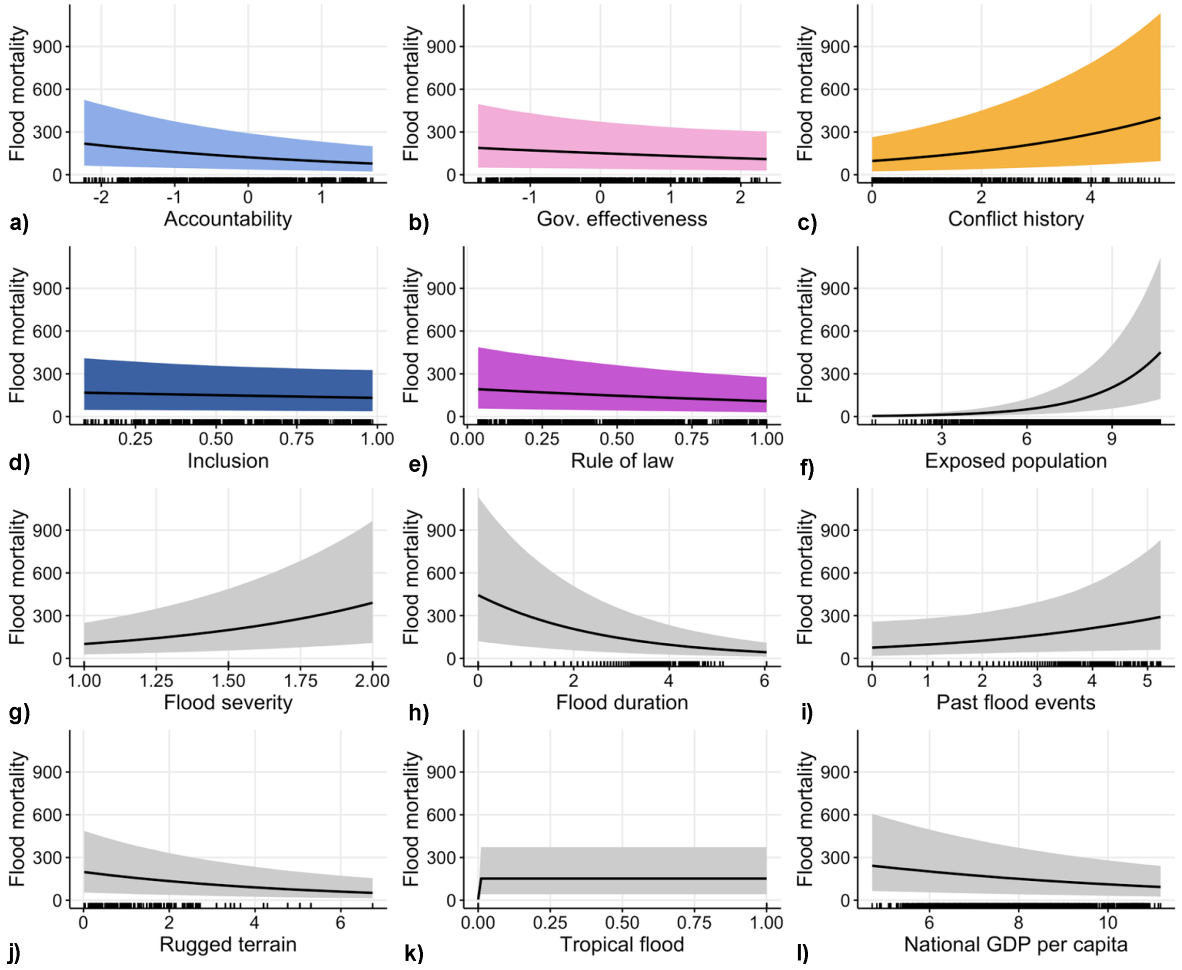

Fig. S14. Conditional effect plots for drivers of flood mortality, excluding local conflict and local HDI. Each plot (a-l) shows the in-sample median of the posterior predictive distribution (black line) and the surrounding 80% predictive interval (shaded area) for the selected indicator, based on 8 × 4,000 Markov chain Monte Carlo draws per model. All models are specified as Bayesian random-effects negative binomial regressions. All effects are computed by including the selected indicator of political development only (color), in addition to the baseline predictors (grey), holding other variables at their observed mean values. Colors reflect the political development dimension of the main predictor in the model (blue: democracy, pink: institutional quality, orange: peace breakdown). Rug plots display the distribution of data points. Plots are shown for a subset of observations excluding the 20% most severe conflict history events, although the underlying models were estimated on the complete training sample, 2000–14 (N=1,914).

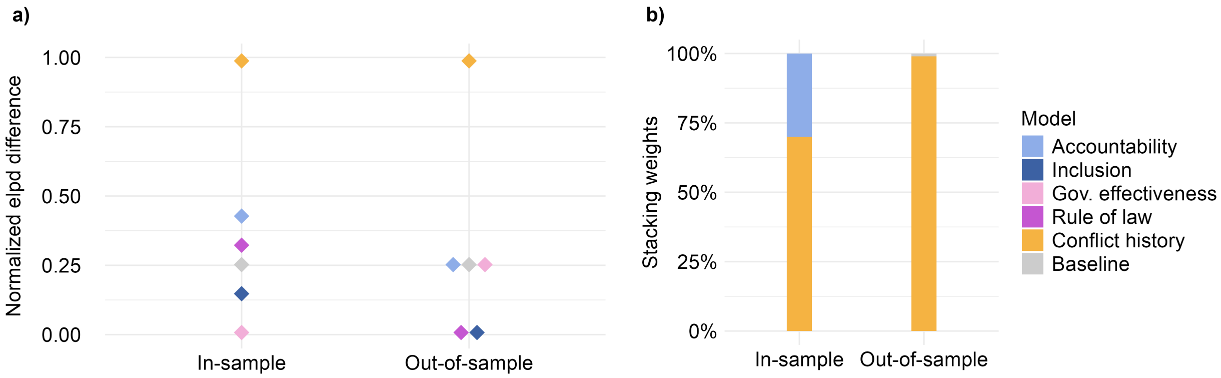

c)

	In-sample (2000-2014)			Out-of-sample (2015-2018)		
Model	elpd LOO	Δelpd	stacking weight	elpd LOO	Δelpd	stacking weight
Conflict history	-4928 (116)	0 (0)	0.7	-597 (37)	0 (0)	0.99
Accountability	-4940 (117)	-11 (8)	0.3	-600 (37)	-3 (1)	0.00
Rule of law	-4942 (117)	-13 (7)	0.0	-601 (37)	-4 (1)	0.00
Baseline	-4942 (117)	-14 (7)	0.0	-600 (37)	-3 (2)	0.01
Inclusion	-4944 (117)	-16 (7)	0.0	-601 (37)	-4 (2)	0.00
Gov. effectiveness	-4948 (118)	-19 (8)	0.0	-600 (37)	-3 (2)	0.00

Fig. S15. Relative predictive performance of political development models on flood mortality, excluding local conflict and local HDI indicators. **a)** In-sample (2000–14, N=1,914) and out-of-sample (2015–18, N=311) expected log predictive density (elpd) for each model, obtained via leave-one-out cross-validation with Pareto-smoothed importance sampling, normalized such that the best-performing model scores 1 and the worst scores 0. **b)** In-sample and out-of-sample stacking weights for each model, reflecting the proportion of observations for which each model provides superior predictive performance. **c)** In-sample and out-of-sample elpd values (non-normalized) and stacking weights for each indicator model, ranked by in-sample elpd performance (values closer to zero indicate better fit; standard errors in parentheses). Each model includes all indicators of a given political development dimension (color) in addition to the baseline (grey). Colors reflect the political development dimension of the main predictor in the model (blue: democracy, pink: institutional quality, orange: peace breakdown).

2.4 Omitting continent-level random effects

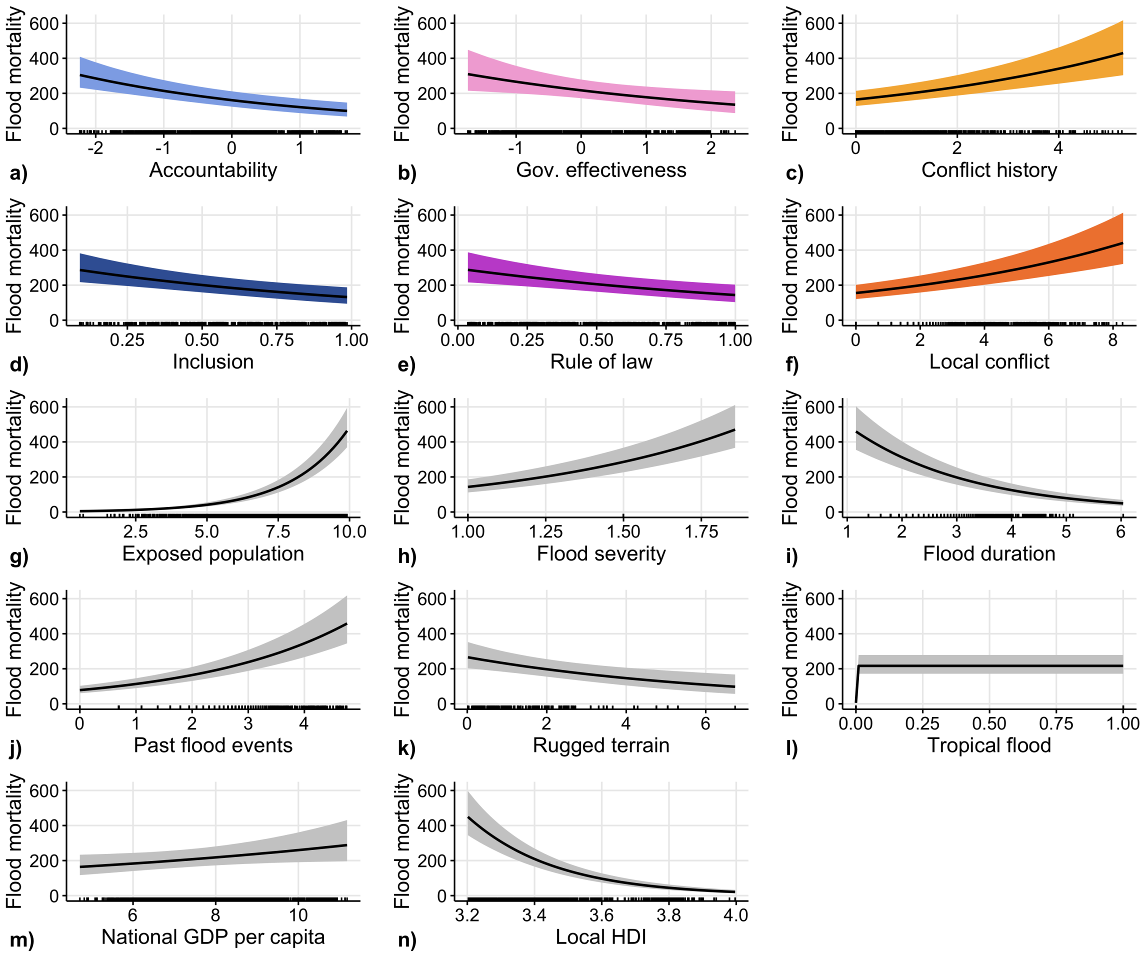

Fig. S16. Conditional effect plots for the models without continent random effects. Each plot (a-n) shows the in-sample median of the posterior predictive distribution (black line) and the surrounding 80% predictive interval (shaded area) for the selected indicator, based on 8 × 4,000 Markov chain Monte Carlo draws per model. The models are specified as Bayesian regressions with linear yearly trend but without continent-level random effects. All effects are computed by including the selected indicator of political development only (color), in addition to the baseline predictors (grey), holding other variables at their observed mean values. Colors reflect the political development dimension of the main predictor in the model (blue: democracy, pink: institutional quality, orange: peace breakdown). Rug plots display the distribution of data points. Plots are shown for a subset of observations excluding the 20% most severe conflict history events, although the underlying models were estimated on the complete training sample, 2000–14 (N=1,914).

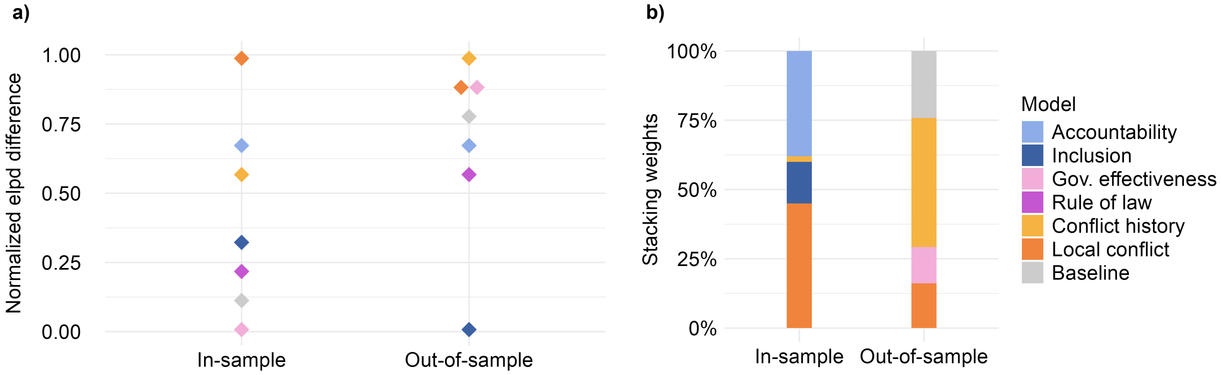

c)

	In-sample (2000-2014)			Out-of-sample (2015-2018)		
Model	elpd LOO	Δelpd	stacking weight	elpd LOO	Δelpd	stacking weight
Local conflict	-4949 (119)	0 (0)	0.45	-603 (37)	-1 (1)	0.16
Accountability	-4952 (118)	-3 (9)	0.38	-604 (38)	-3 (1)	0.00
Conflict history	-4953 (119)	-4 (5)	0.02	-602 (37)	0 (0)	0.46
Inclusion	-4955 (118)	-6 (9)	0.15	-611 (37)	-9 (2)	0.00
Rule of law	-4956 (118)	-7 (8)	0.00	-605 (38)	-4 (1)	0.00
Baseline	-4958 (119)	-8 (7)	0.00	-603 (37)	-2 (1)	0.24
Gov. effectiveness	-4958 (119)	-9 (7)	0.00	-603 (37)	-1 (1)	0.13

Fig. S17. Relative predictive performance of political development models on flood mortality excluding continent-level random effects. a) In-sample (2000–14, N=1,914) and out-of-sample (2015–18, N=311) expected log predictive density (elpd) for each model, obtained via leave-one-out cross-validation with Pareto-smoothed importance sampling, normalized such that the best-performing model scores 1 and the worst scores 0. **b)** In-sample and out-of-sample stacking weights for each model, reflecting the proportion of observations for which each model provides superior predictive performance. **c)** In-sample and out-of-sample elpd values (non-normalized) and stacking weights for each indicator model, ranked by in-sample elpd performance (values closer to zero indicate better fit; standard errors in parentheses). Each model includes all indicators of a given political development dimension (color) in addition to the baseline (grey). Colors reflect the political development dimension of the main predictor in the model (blue: democracy, pink: institutional quality, orange: peace breakdown).

2.5 Omitting yearly trends

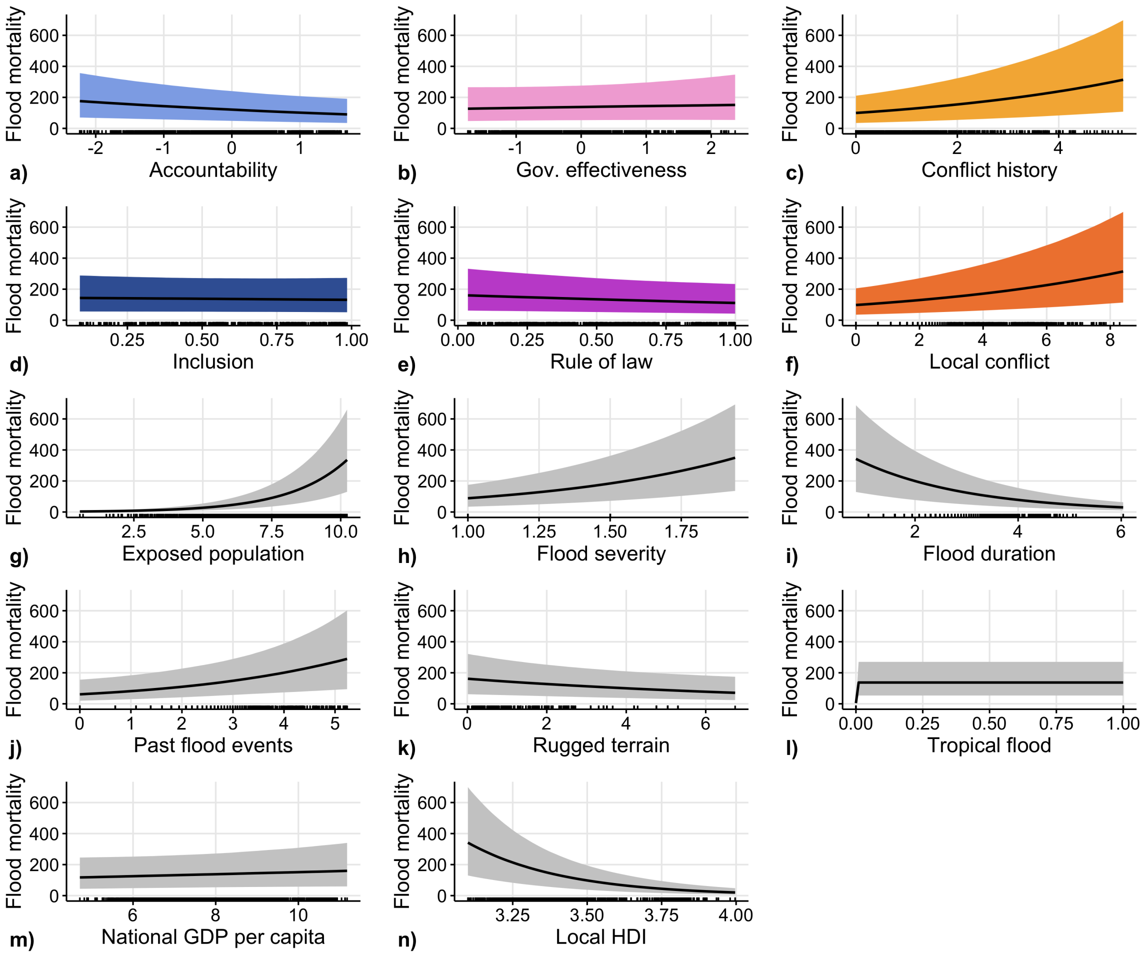

Fig. S18. Conditional effect plots for the models without yearly trends. Each plot (a-n) shows the in-sample median of the posterior predictive distribution (black line) and the surrounding 80% predictive interval (shaded area) for the selected indicator, based on 8 × 4,000 Markov chain Monte Carlo draws per model. All models are specified as Bayesian random-effects negative binomial regressions, but without linear yearly trend. All effects are computed by including the selected indicator of political development only (color), in addition to the baseline predictors (grey), holding other variables at their observed mean values. Colors reflect the political development dimension of the main predictor in the model (blue: democracy, pink: institutional quality, orange: peace breakdown). Rug plots display the distribution of data points. Plots are shown for a subset of observations excluding the 20% most severe conflict history events, although the underlying models were estimated on the complete training sample, 2000–14 (N=1,914).

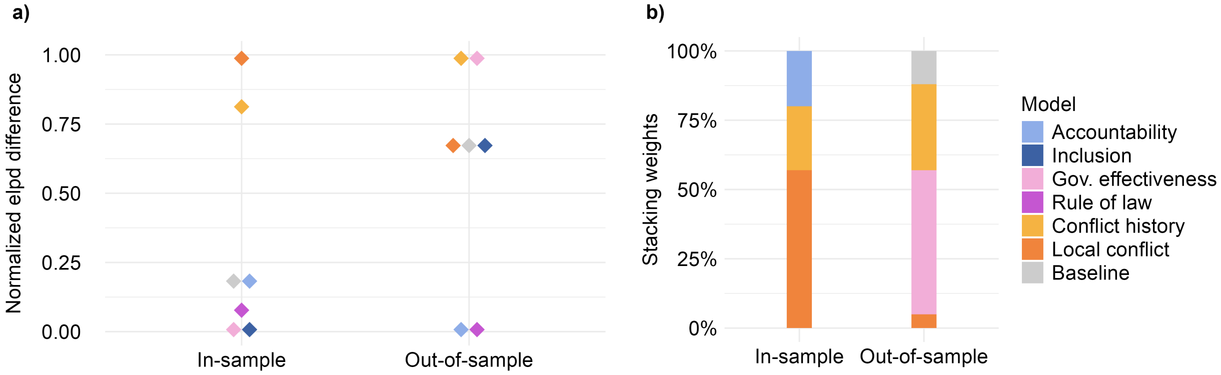

c)

	In-sample (2000-2014)			Out-of-sample (2015-2018)		
Model	elpd LOO	Δelpd	stacking weight	elpd LOO	Δelpd	stacking weight
Local conflict	-4920 (117)	0 (0)	0.57	-608 (36)	-1 (1)	0.05
Conflict history	-4923 (117)	-3 (5)	0.23	-607 (37)	0 (1)	0.31
Baseline	-4932 (118)	-12 (7)	0.00	-608 (37)	-1 (0)	0.12
Accountability	-4932 (118)	-12 (8)	0.20	-610 (37)	-3 (1)	0.00
Rule of law	-4933 (118)	-14 (7)	0.00	-610 (37)	-3 (0)	0.00
Gov. effectiveness	-4935 (119)	-15 (7)	0.00	-607 (37)	0 (0)	0.52
Inclusion	-4935 (118)	-15 (8)	0.00	-608 (37)	-1 (0)	0.00

Fig. S19. Relative predictive performance of political development models on flood mortality, excluding yearly trends. a) In-sample (2000–14, N=1,914) and out-of-sample (2015–18, N=311) expected log predictive density (elpd) for each model, obtained via leave-one-out cross-validation with Pareto-smoothed importance sampling, normalized such that the best-performing model scores 1 and the worst scores 0. **b)** In-sample and out-of-sample stacking weights for each model, reflecting the proportion of observations for which each model provides superior predictive performance. **c)** In-sample and out-of-sample elpd values (non-normalized) and stacking weights for each indicator model, ranked by in-sample elpd performance (values closer to zero indicate better fit; standard errors in parentheses). Each model includes all indicators of a given political development dimension (color) in addition to the baseline (grey). Colors reflect the political development dimension of the main predictor in the model (blue: democracy, pink: institutional quality, orange: peace breakdown).

2.6 Flood exposure at least 1,000 people

The full sample (N=2,225) contains many small flood events. To assess whether the models perform equally well on larger floods, we estimated the models on a sample restricted to floods that exposed at least 1,000 people (N=1,528, full sample). The results are comparable to those reported in the article.

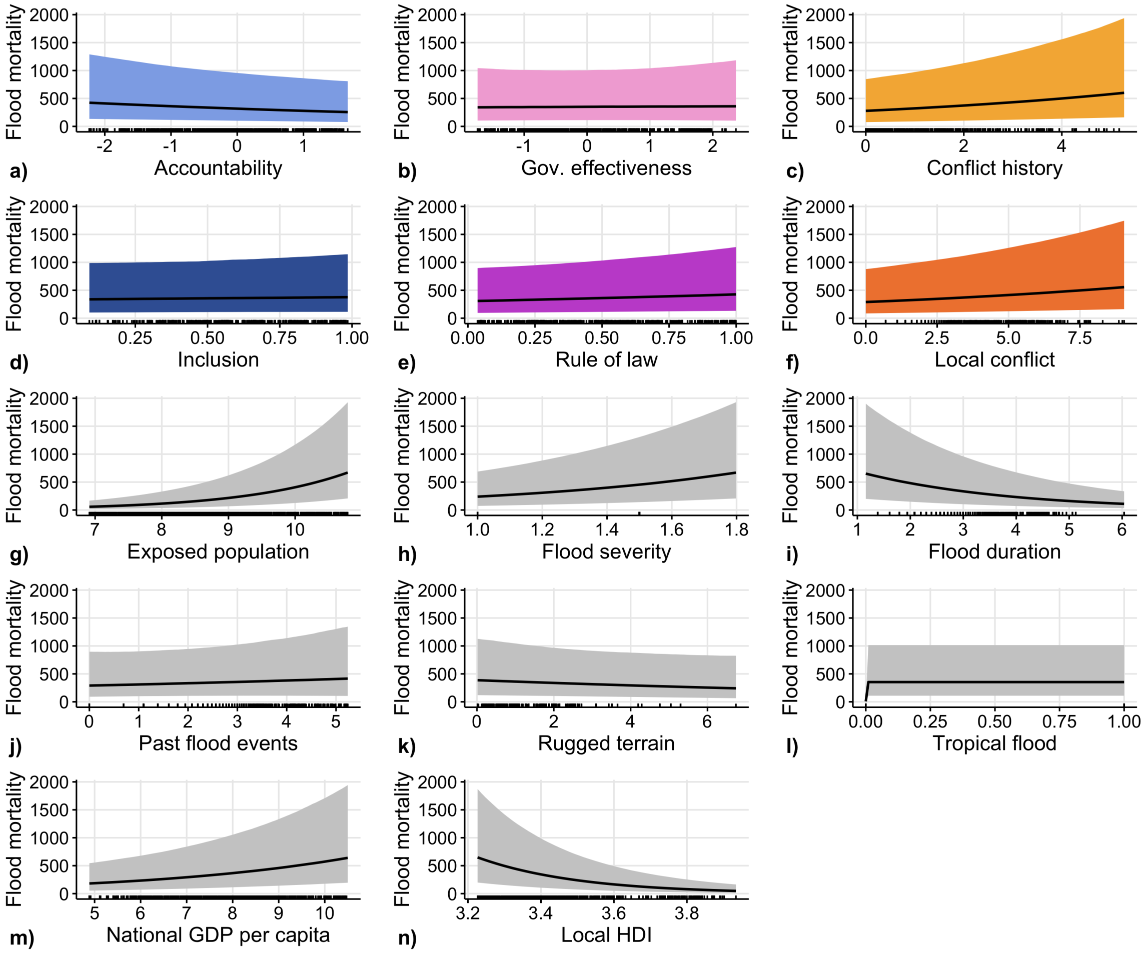

Fig. S20. Conditional effect plots for drivers of flood mortality for floods exposing at least 1,000 people. Each plot (a-n) shows the in-sample median of the posterior predictive distribution (black line) and the surrounding 80% predictive interval (shaded area) for the selected indicator, based on 8 × 4,000 Markov chain Monte Carlo draws per model. All models are specified as Bayesian random-effects negative binomial regressions. All effects are computed by including the selected indicator of political development only (color), in addition to the baseline predictors (grey), holding other variables at their observed mean values. Colors reflect the political development dimension of the main predictor in the model (blue: democracy, pink: institutional quality, orange: peace breakdown). Rug plots display the distribution of data points. Plots are shown for a subset of observations excluding the 20% most severe conflict history events. The training sample is restricted to flood events that affected at least 1,000 people in 2000-2014 (N=1,323).

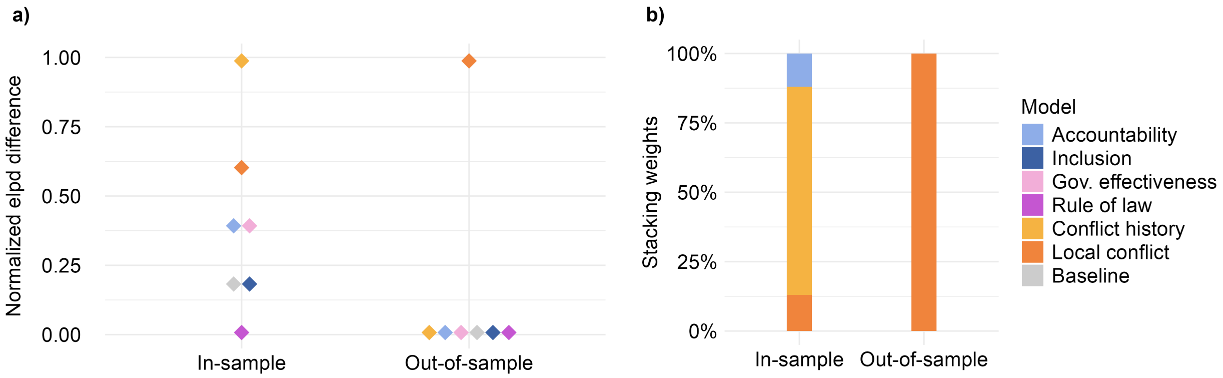

c)

	In-sample (2000-2014)			Out-of-sample (2015-2018)		
Model	elpd LOO	Δelpd	stacking weight	elpd LOO	Δelpd	stacking weight
Conflict history	-4099 (99)	0 (0)	0.75	-529 (32)	-1 (1)	0
Local conflict	-4101 (100)	-2 (3)	0.13	-528 (32)	0 (0)	1
Accountability	-4102 (100)	-3 (3)	0.12	-530 (32)	-1 (1)	0
Gov. effectiveness	-4102 (100)	-3 (3)	0.00	-530 (32)	-1 (1)	0
Baseline	-4103 (100)	-4 (3)	0.00	-529 (32)	-1 (1)	0
Inclusion	-4103 (100)	-4 (3)	0.00	-529 (32)	-1 (1)	0
Rule of law	-4104 (100)	-5 (4)	0.00	-529 (32)	-1 (1)	0

Fig. S21. Relative predictive performance of political development models on flood mortality. The sample is limited to flood events exposing at least 1,000 people. a) In-sample (2000–14, N=1,323) and out-of-sample (2015–18, N=205) expected log predictive density (elpd) for each model, obtained via leave-one-out cross-validation with Pareto-smoothed importance sampling, normalized such that the best-performing model scores 1 and the worst scores 0. **b)** In-sample and out-of-sample stacking weights for each model, reflecting the proportion of observations for which each model provides superior predictive performance. **c)** In-sample and out-of-sample elpd values (non-normalized) and stacking weights for each indicator model, ranked by in-sample elpd performance (values closer to zero indicate better fit; standard errors in parentheses). Each model includes all indicators of a given political development dimension (color) in addition to the baseline (grey). Colors reflect the political development dimension of the main predictor in the model (blue: democracy, pink: institutional quality, orange: peace breakdown).

2.7 Deadly floods

Likewise, the full sample (N=2,225) contains many non-fatal floods. As shown below, the results remain substantively unaffected by restricting the analytical sample to deadly floods (N=1,070, full sample).

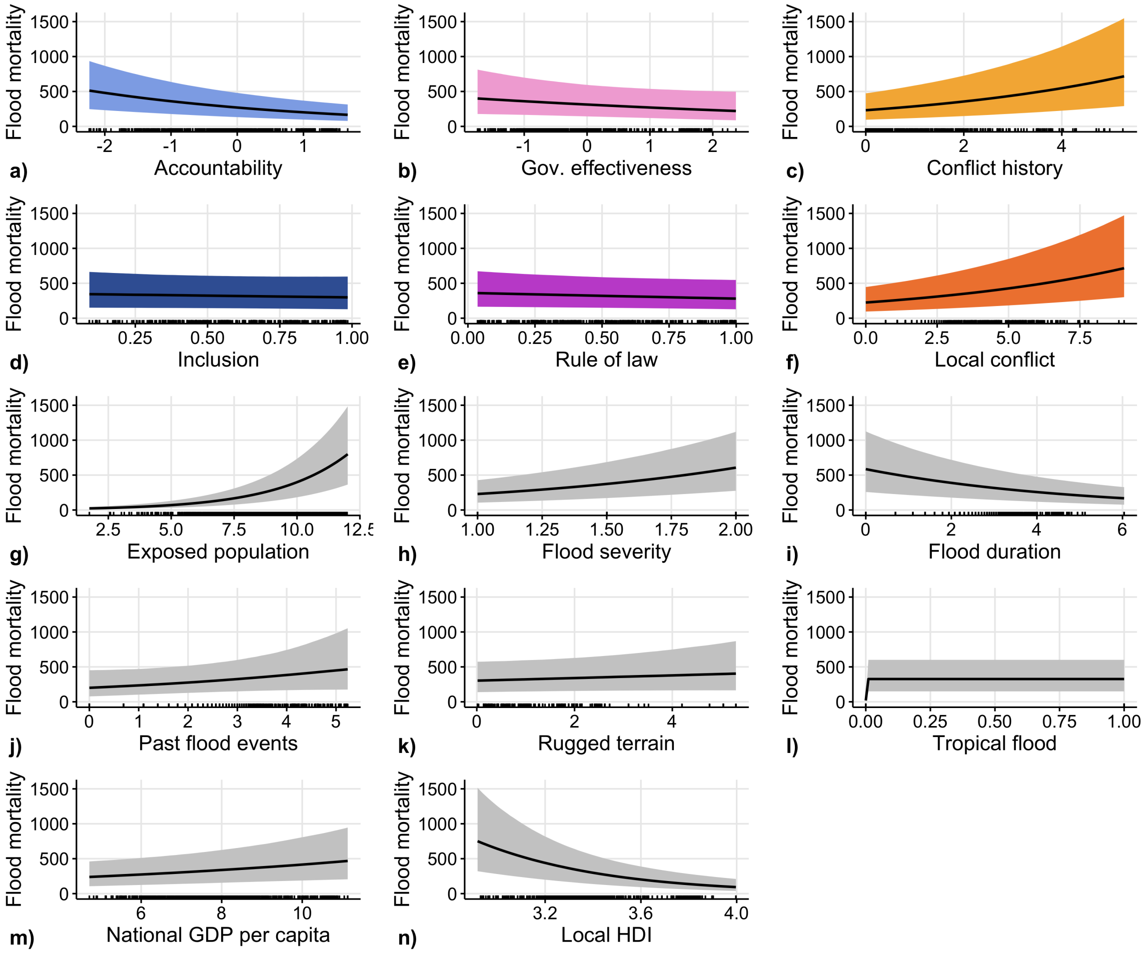

Fig. S22. Conditional effect plots for drivers of flood mortality for deadly floods. Each plot (a-n) shows the in-sample median of the posterior predictive distribution (black line) and the surrounding 80% predictive interval (shaded area) for the selected indicator, based on 8 × 4,000 Markov chain Monte Carlo draws per model. All models are specified as Bayesian random-effects negative binomial regressions. All effects are computed by including the selected indicator of political development only (color), in addition to the baseline predictors (grey), holding other variables at their observed mean values. Colors reflect the political development dimension of the main predictor in the model (blue: democracy, pink: institutional quality, orange: peace breakdown). Rug plots display the distribution of data points. Plots are shown for a subset of observations excluding the 20% most severe conflict history events. The underlying models were estimated on a training sample limited to deadly floods, 2000–14 (N=957).

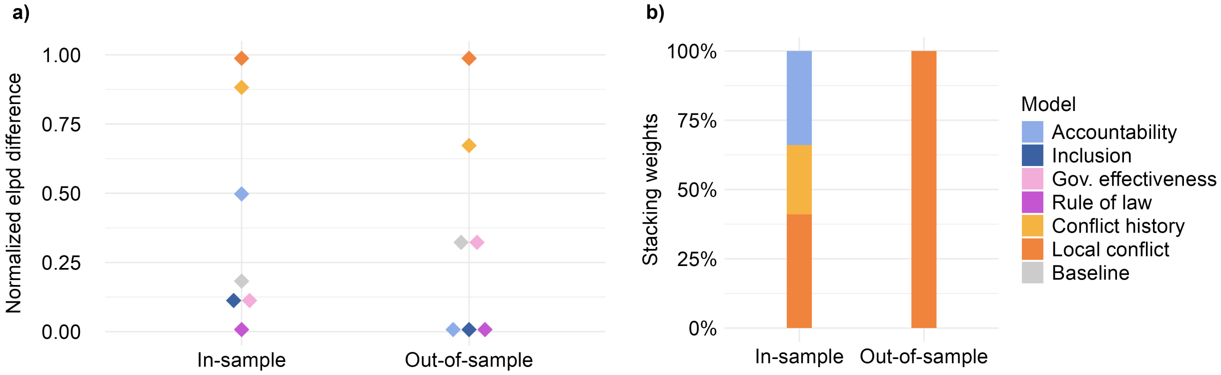

c)

	In-sample (2000-2014)			Out-of-sample (2015-2018)		
Model	elpd LOO	Δelpd	stacking weight	elpd LOO	Δelpd	stacking weight
Local conflict	-4150 (87)	0 (0)	0.41	-473 (16)	0 (0)	1
Conflict history	-4152 (85)	-2 (6)	0.25	-474 (16)	-1 (1)	0
Accountability	-4158 (87)	-8 (9)	0.34	-476 (16)	-3 (2)	0
Baseline	-4163 (88)	-13 (8)	0.00	-475 (16)	-2 (1)	0
Inclusion	-4164 (88)	-14 (7)	0.00	-475 (16)	-3 (1)	0
Gov. effectiveness	-4164 (88)	-14 (8)	0.00	-475 (16)	-2 (1)	0
Rule of law	-4166 (89)	-16 (8)	0.00	-476 (16)	-3 (1)	0

Fig. S23. Relative predictive performance of political development models on flood mortality on sample limited to deadly floods. a) In-sample (2000–14, N=957) and out-of-sample (2015–18, N=113) expected log predictive density (elpd) for each model, obtained via leave-one-out cross-validation with Pareto-smoothed importance sampling, normalized such that the best-performing model scores 1 and the worst scores 0. **b)** In-sample and out-of-sample stacking weights for each model, reflecting the proportion of observations for which each model provides superior predictive performance. **c)** In-sample and out-of-sample elpd values (non-normalized) and stacking weights for each indicator model, ranked by in-sample elpd performance (values closer to zero indicate better fit; standard errors in parentheses). Each model includes all indicators of a given political development dimension (color) in addition to the baseline (grey). Colors reflect the political development dimension of the main predictor in the model (blue: democracy, pink: institutional quality, orange: peace breakdown).

2.8 Alternative training/test split

Floods are a recurring phenomenon in many parts of the world, but the magnitude and impact of flood events vary substantially across space and time. For this reason, the results presented in the article can be sensitive to the cut-off year chosen to split the training and test samples. Here, we show results for alternative models trained for a shorter 2000–10 sample and evaluated on a longer 2011–18 test sample. The results mirror those reported in the main article.

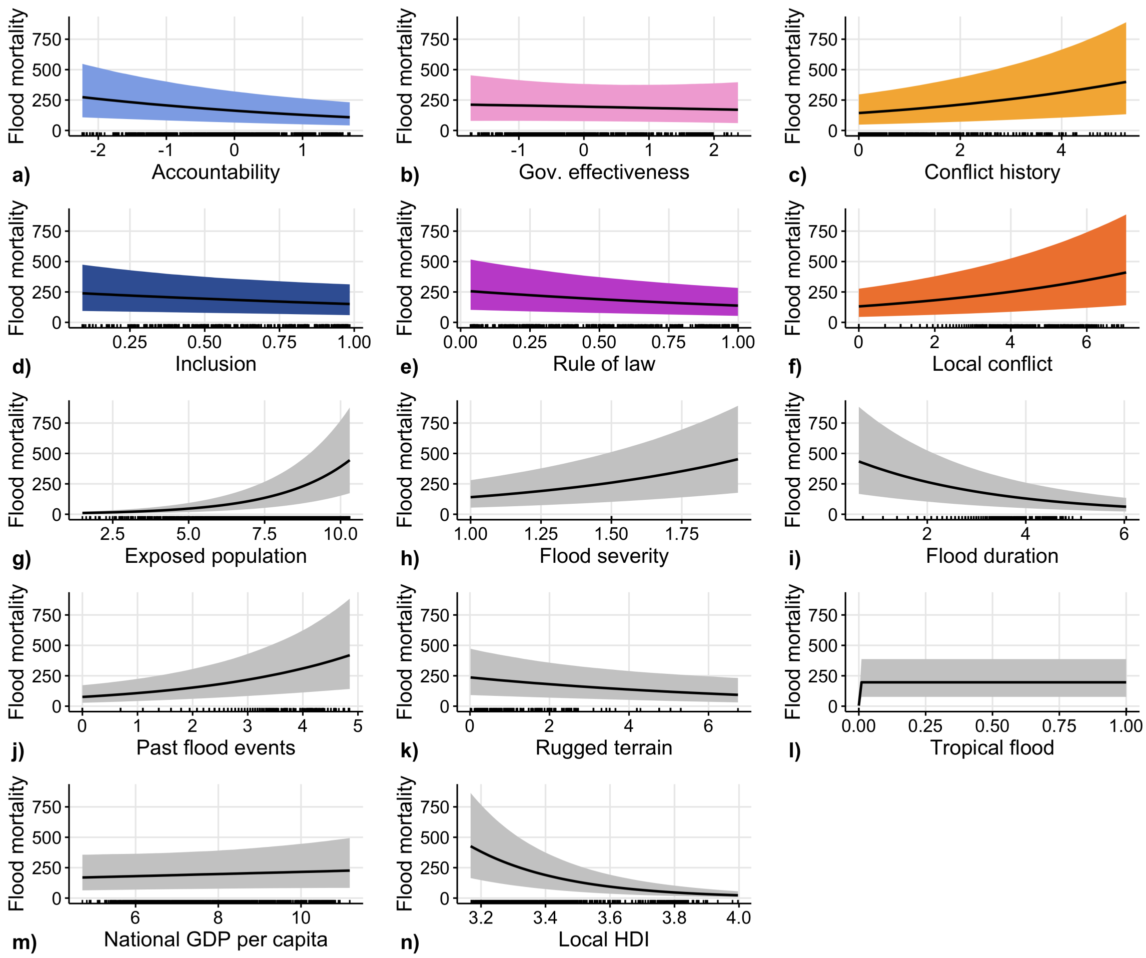

Fig. S24. Conditional effect plots for drivers of flood mortality, using alternative periods for training and test samples. Each plot (a-n) shows the in-sample median of the posterior predictive distribution (black line) and the surrounding 80% predictive interval (shaded area) for the selected indicator, based on 8 × 4,000 Markov chain Monte Carlo draws per model. All models are specified as Bayesian random-effects negative binomial regressions. All effects are computed by including the selected indicator of political development only (color), in addition to the baseline predictors (grey), holding other variables at their observed mean values. Colors reflect the political development dimension of the main predictor in the model (blue: democracy, pink: institutional quality, orange: peace breakdown). Rug plots display the distribution of data points. Plots are shown for a subset of observations excluding the 20% most severe conflict history events. The underlying models were estimated on an alternative training sample for 2000–10 (N=1,559).

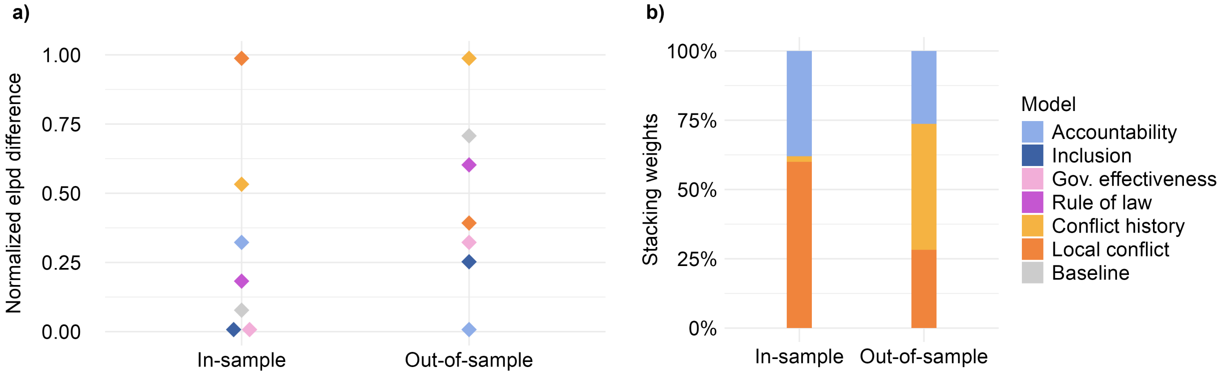

c)

	In-sample (2000-2010)			Out-of-sample (2011-2018)		
Model	elpd LOO	Δelpd	stacking weight	elpd LOO	Δelpd	stacking weight
Local conflict	-3993 (103)	0 (0)	0.60	-1625 (131)	-13 (12)	0.28
Conflict history	-4000 (103)	-7 (5)	0.02	-1612 (120)	0 (0)	0.45
Accountability	-4003 (103)	-10 (8)	0.38	-1633 (137)	-21 (19)	0.26
Rule of law	-4006 (104)	-12 (7)	0.00	-1620 (123)	-8 (4)	0.00
Baseline	-4007 (105)	-14 (7)	0.00	-1618 (121)	-6 (3)	0.00
Inclusion	-4008 (105)	-15 (8)	0.00	-1628 (129)	-16 (11)	0.00
Gov. effectiveness	-4009 (105)	-15 (7)	0.00	-1626 (129)	-14 (10)	0.00

Fig. S25. Relative predictive performance of political development models on flood mortality with alternative training and test sets. The training and test sets are split using 2010 as the cutoff as opposed to 2014 used in the main specifications. **a)** In-sample (2000–10, N=1,559) and out-of-sample (2011–18, N=666) expected log predictive density (elpd) for each model, obtained via leave-one-out cross-validation with Pareto-smoothed importance sampling, normalized such that the best-performing model scores 1 and the worst scores 0. **b)** In-sample and out-of-sample stacking weights for each model, reflecting the proportion of observations for which each model provides superior predictive performance. **c)** In-sample and out-of-sample elpd values (non-normalized) and stacking weights for each indicator model, ranked by in-sample elpd performance (values closer to zero indicate better fit; standard errors in parentheses). Each model includes all indicators of a given political development dimension (color) in addition to the baseline (grey). Colors reflect the political development dimension of the main predictor in the model (blue: democracy, pink: institutional quality, orange: peace breakdown).

2.9 Random training/test split

Here, we show the results for alternative models where training and test partition are split using a random 80–20% partitioning. This is equivalent to a thought experiment where we assess how the models would perform if the predictors were stable, and without accounting for temporal dependencies. The results mirror those reported in the main article.

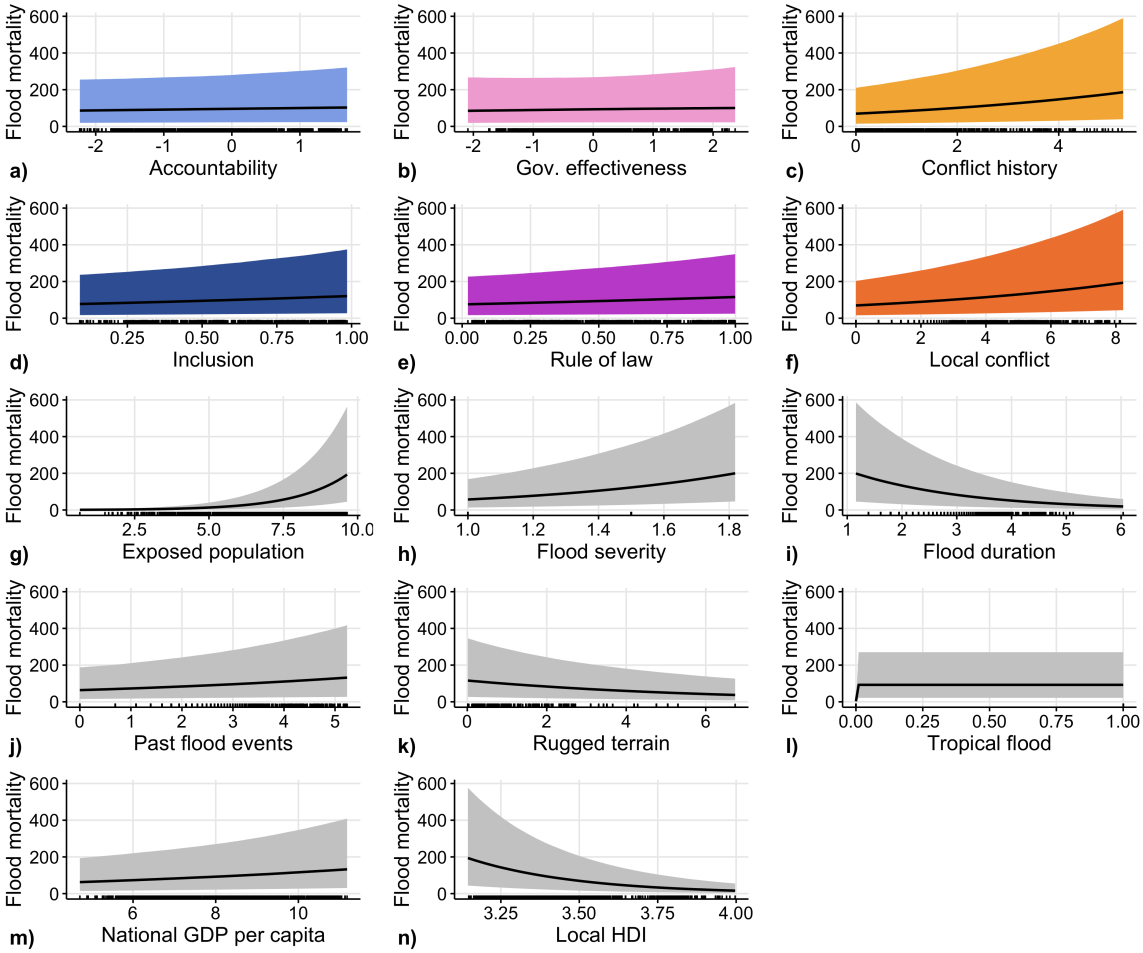

Fig. S26. Conditional effect plots for drivers of flood mortality, using a random 80–20% split for training and test samples. Each plot (a-n) shows the in-sample median of the posterior predictive distribution (black line) and the surrounding 80% predictive interval (shaded area) for the selected indicator, based on 8 × 4,000 Markov chain Monte Carlo draws per model. All models are specified as Bayesian random-effects negative binomial regressions. All effects are computed by including the selected indicator of political development only (color), in addition to the baseline predictors (grey), holding other variables at their observed mean values. Colors reflect the political development dimension of the main predictor in the model (blue: democracy, pink: institutional quality, orange: peace breakdown). Rug plots display the distribution of data points. Plots are shown for a subset of observations excluding the 20% most severe conflict history events. The underlying models were estimated on an alternative training sample representing a random 20% portion of the full dataset (N=1,780).

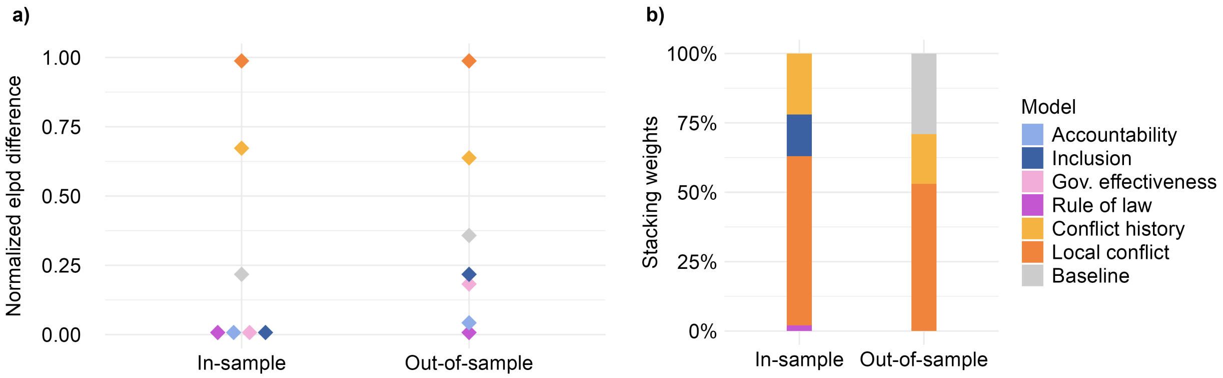

c)

	In-sample			Out-of-sample		
Model	elpd LOO	Δelpd	stacking weight	elpd LOO	Δelpd	stacking weight
Local conflict	-4363 (111)	0 (0)	0.61	-1239 (113)	0 (0)	0.53
Conflict history	-4366 (111)	-3 (5)	0.22	-1256 (128)	-17 (20)	0.18
Baseline	-4370 (111)	-7 (6)	0.00	-1269 (137)	-29 (30)	0.29
Rule of law	-4372 (112)	-9 (7)	0.02	-1285 (146)	-46 (38)	0.00
Accountability	-4372 (112)	-9 (7)	0.00	-1283 (145)	-44 (37)	0.00
Gov. effectiveness	-4373 (112)	-9 (6)	0.00	-1278 (144)	-38 (36)	0.00
Inclusion	-4373 (112)	-9 (7)	0.15	-1275 (138)	-36 (29)	0.00

Fig. S27. Relative predictive performance of political development models on flood mortality with alternative training and test sets. The training and test sets are split using a random 80–20% split, as opposed to the temporal split used in the main specifications. **a)** In-sample (N=1,780) and out-of-sample (N=445) expected log predictive density (elpd) for each model, obtained via leave-one-out cross-validation with Pareto-smoothed importance sampling, normalized such that the best-performing model scores 1 and the worst scores 0. **b)** In-sample and out-of-sample stacking weights for each model, reflecting the proportion of observations for which each model provides superior predictive performance. **c)** In-sample and out-of-sample elpd values (non-normalized) and stacking weights for each indicator model, ranked by in-sample elpd performance (values closer to zero indicate better fit; standard errors in parentheses). Each model includes all indicators of a given political development dimension (color) in addition to the baseline (grey). Colors reflect the political development dimension of the main predictor in the model (blue: democracy, pink: institutional quality, orange: peace breakdown).

2.10 Excluding influential data points

To further gauge the robustness of our results we estimate separate models excluding influential data points and the most flood-affected countries – Myanmar, India, China and Bangladesh, respectively.

2.10.1 Excluding the most severe flood event

The most severe flood-country event in the full sample is the flooding in Myanmar in association with Cyclone Nargis in May 2008, which is estimated in our data to have claimed shy of 80,000 lives within the boundaries of the country. The next most severe event, the earthquake-generated tsunami in Japan in March 2011, generated only a fraction of that number (10,000 fatalities). As shown in the figures below, the main findings are robust to excluding the Nargis flood event.

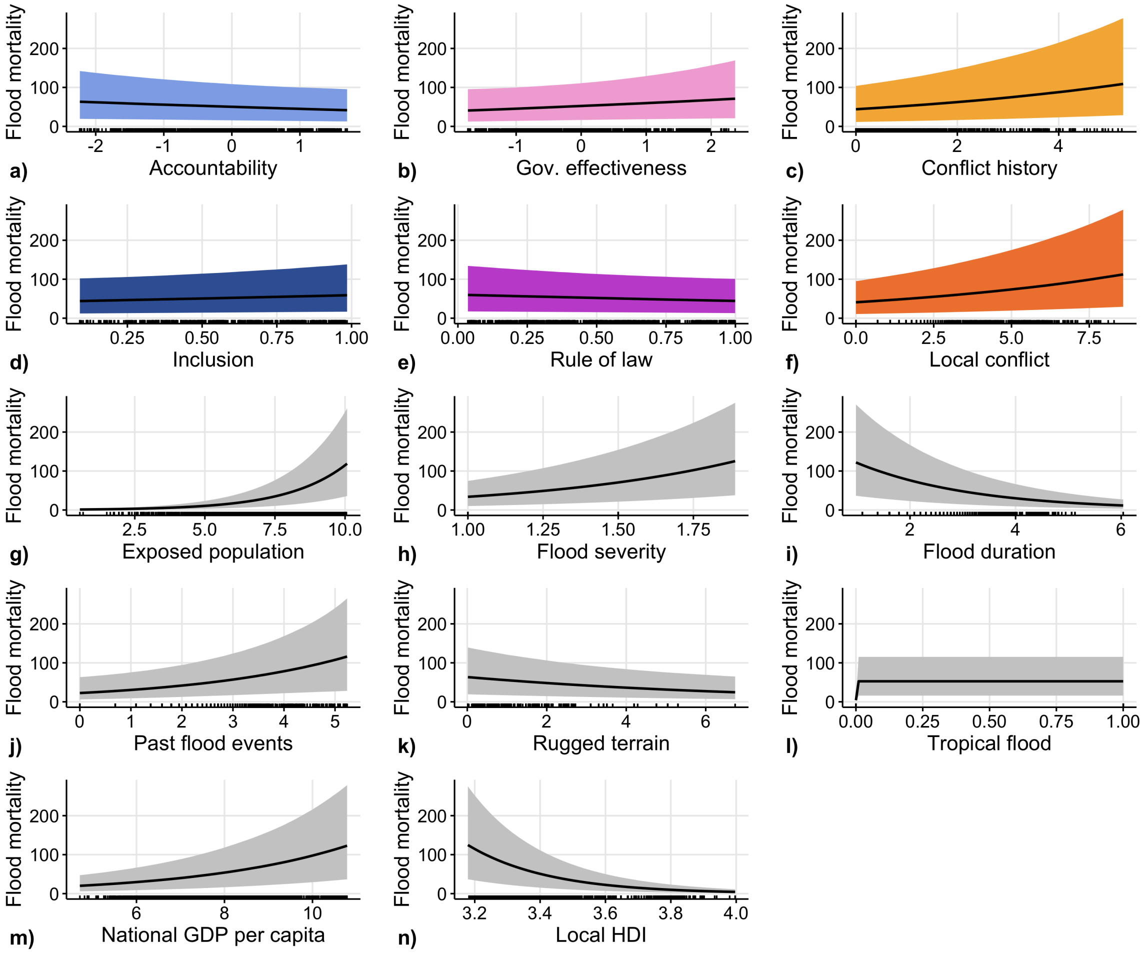

Fig. S28. Conditional effect plots for drivers of flood mortality, excluding the most extreme outlier. Each plot (a-n) shows the in-sample median of the posterior predictive distribution (black line) and the surrounding 80% predictive interval (shaded area) for the selected indicator, based on 8 × 4,000 Markov chain Monte Carlo draws per model. All models are specified as Bayesian random-effects negative binomial regressions. All effects are computed by including the selected indicator of political development only (color), in addition to the baseline predictors (grey), holding other variables at their observed mean values. Colors reflect the political development dimension of the main predictor in the model (blue: democracy, pink: institutional quality, orange: peace breakdown). Rug plots display the distribution of data points. Plots are shown for a subset of observations excluding the 20% most severe conflict history events. The underlying models were estimated on a restricted training sample excluding the most extreme outlier, 2000–14 (N=1,913).

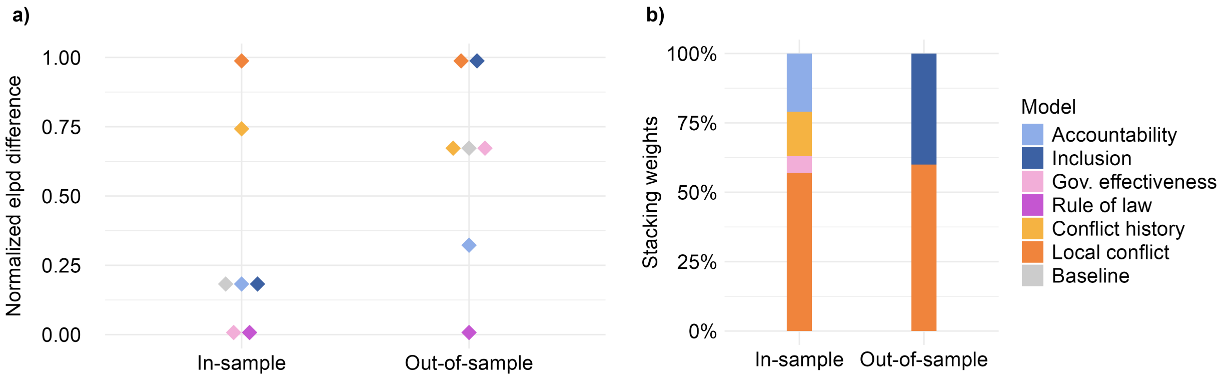

c)

	In-sample (2000-2014)			Out-of-sample (2015-2018)		
Model	elpd LOO	Δelpd	stacking weight	elpd LOO	Δelpd	stacking weight
Local conflict	-4872 (113)	0 (0)	0.57	-589 (38)	0 (0)	0.6
Conflict history	-4876 (113)	-3 (5)	0.16	-590 (38)	-1 (1)	0.0
Baseline	-4881 (114)	-9 (7)	0.00	-590 (38)	-1 (2)	0.0
Accountability	-4882 (114)	-9 (7)	0.21	-591 (38)	-2 (2)	0.0
Inclusion	-4882 (114)	-9 (7)	0.00	-589 (38)	0 (2)	0.4
Gov. effectiveness	-4883 (114)	-11 (8)	0.06	-590 (38)	-1 (2)	0.0
Rule of law	-4883 (114)	-11 (7)	0.00	-591 (38)	-3 (1)	0.0

Fig. S29. Relative predictive performance of political development models on flood mortality. The sample (N=1,913) excludes an outlier for flood mortality, causing 79,828 deaths. a) In-sample (2000–14, N=1,913) and out-of-sample (2015–18, N=311) expected log predictive density (elpd) for each model, obtained via leave-one-out cross-validation with Pareto-smoothed importance sampling, normalized such that the best-performing model scores 1 and the worst scores 0. **b)** In-sample and out-of-sample stacking weights for each model, reflecting the proportion of observations for which each model provides superior predictive performance. **c)** In-sample and out-of-sample elpd values (non-normalized) and stacking weights for each indicator model, ranked by in-sample elpd performance (values closer to zero indicate better fit; standard errors in parentheses). Each model includes all indicators of a given political development dimension (color) in addition to the baseline (grey). Colors reflect the political development dimension of the main predictor in the model (blue: democracy, pink: institutional quality, orange: peace breakdown).

2.10.2 Excluding Myanmar

The most severely flood-affected country in the GFD data in terms of high frequency of flood events and high average mortality is Myanmar (N=53; avg. mortality=1,512). The following figures show results estimates on a sample that excludes Myanmar.

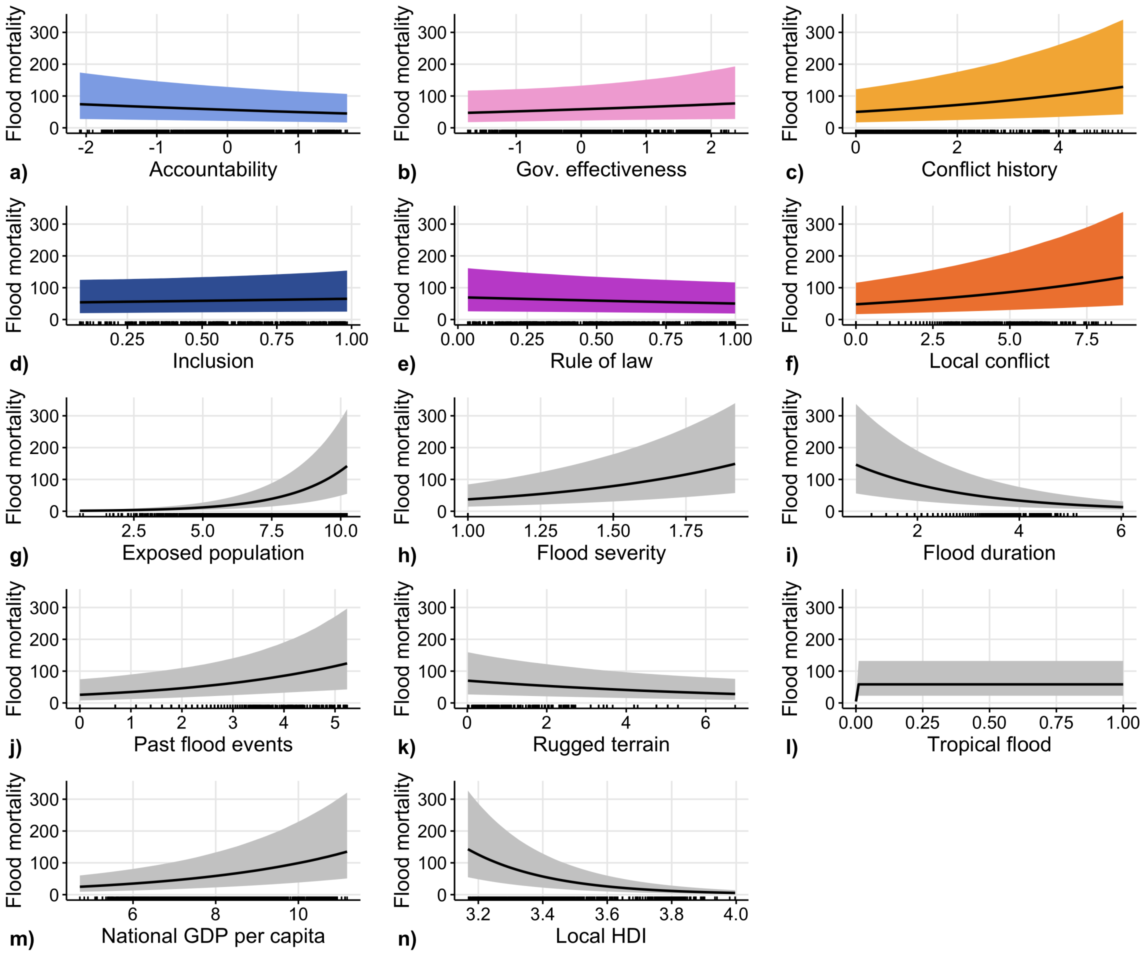

Fig. S30. Conditional effect plots for drivers of flood mortality, excluding observations for Myanmar. Each plot (a-n) shows the in-sample median of the posterior predictive distribution (black line) and the surrounding 80% predictive interval (shaded area) for the selected indicator, based on 8 $\times$ 4,000 Markov chain Monte Carlo draws per model. All models are specified as Bayesian random-effects negative binomial regressions. All effects are computed by including the selected indicator of political development only (color), in addition to the baseline predictors (grey), holding other variables at their observed mean values. Colors reflect the political development dimension of the main predictor in the model (blue: democracy, pink: institutional quality, orange: peace breakdown). Rug plots display the distribution of data points. Plots are shown for a subset of observations excluding the 20% most severe conflict history events. The underlying models were estimated on a restricted training sample that excludes Myanmar, 2000–14 (N=1,871).

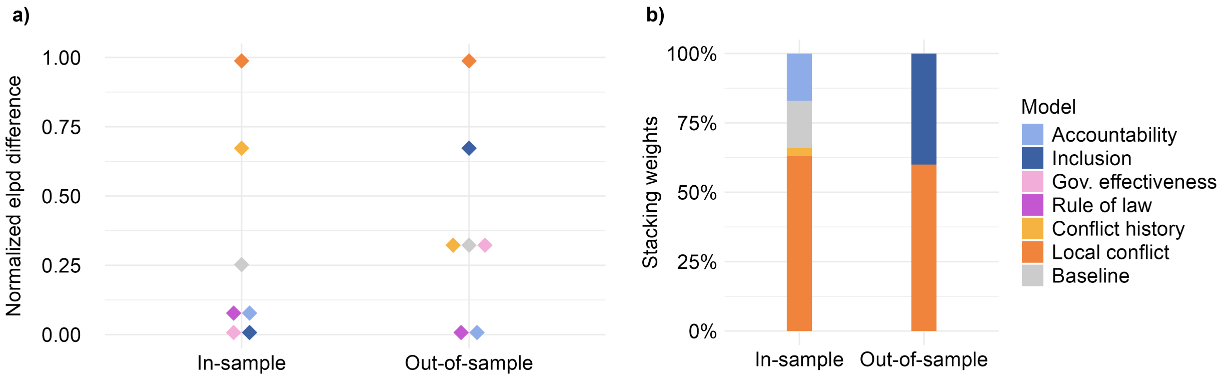

c)

	In-sample (2000-2014)			Out-of-sample (2015-2018)		
Model	elpd LOO	Δelpd	stacking weight	elpd LOO	Δelpd	stacking weight
Local conflict	-4791 (112)	0 (0)	0.63	-567 (37)	0 (0)	0.6
Conflict history	-4795 (112)	-4 (5)	0.03	-569 (38)	-2 (1)	0.0
Baseline	-4799 (113)	-9 (7)	0.17	-568 (37)	-2 (2)	0.0
Rule of law	-4801 (113)	-11 (7)	0.00	-570 (37)	-3 (1)	0.0
Accountability	-4802 (113)	-11 (7)	0.17	-570 (37)	-3 (1)	0.0
Gov. effectiveness	-4802 (113)	-12 (7)	0.00	-569 (37)	-2 (2)	0.0
Inclusion	-4803 (113)	-12 (7)	0.00	-568 (37)	-1 (2)	0.4

Fig. S31. Relative predictive performance of political development models on flood mortality, excluding observations for Myanmar. The sample excludes flood events occurring in Myanmar, the country with highest flood mortality in our data. **a)** In-sample (2000–14, N=1,871) and out-of-sample (2015–18, N=301) expected log predictive density (elpd) for each model, obtained via leave-one-out cross-validation with Pareto-smoothed importance sampling, normalized such that the best-performing model scores 1 and the worst scores 0. **b)** In-sample and out-of-sample stacking weights for each model, reflecting the proportion of observations for which each model provides superior predictive performance. **c)** In-sample and out-of-sample elpd values (non-normalized) and stacking weights for each indicator model, ranked by in-sample elpd performance (values closer to zero indicate better fit; standard errors in parentheses). Each model includes all indicators of a given political development dimension (color) in addition to the baseline (grey). Colors reflect the political development dimension of the main predictor in the model (blue: democracy, pink: institutional quality, orange: peace breakdown).

2.10.3 Excluding India

Another frequently flood-exposed country is India, with 145 events recorded for the 2000–18 period (avg. mortality per event=113.32). The following figures show results without India.

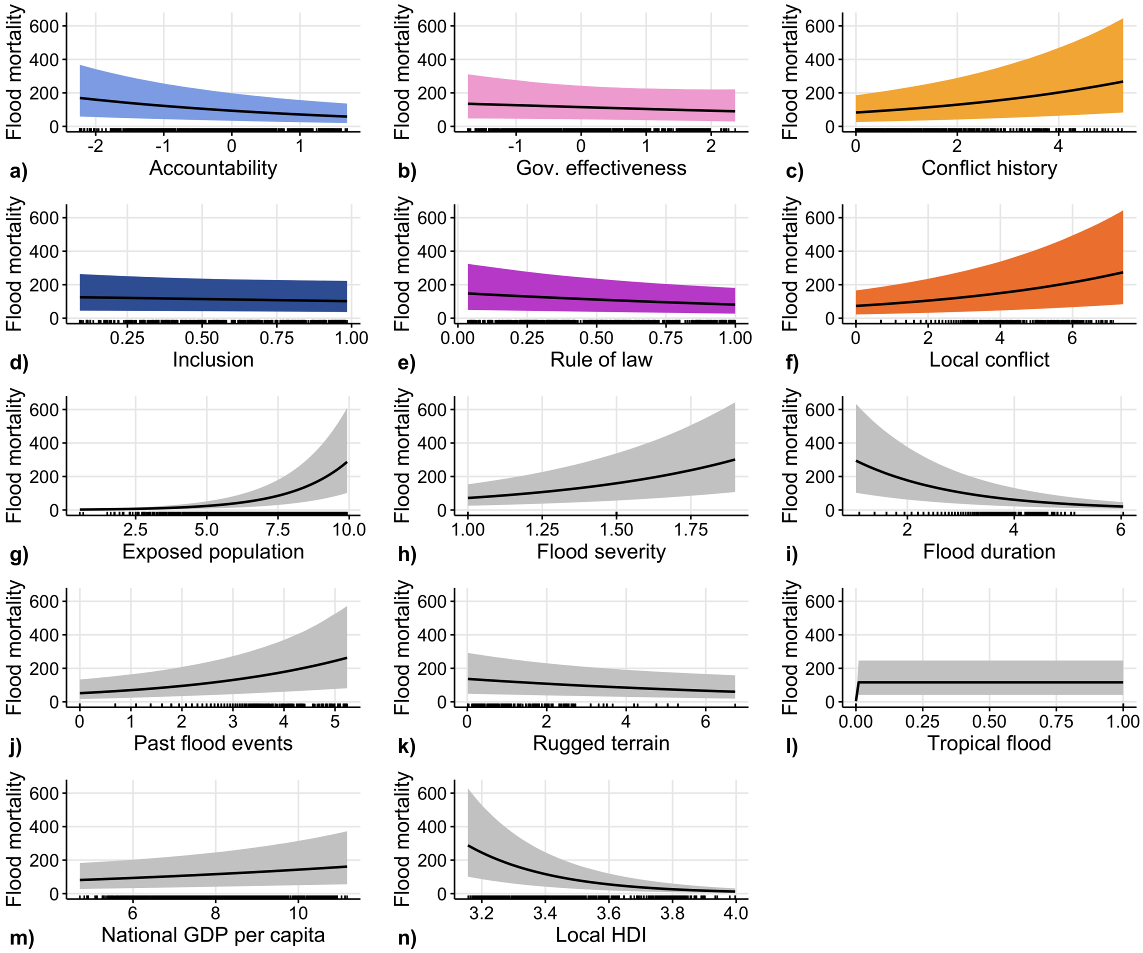

Fig. S32. Conditional effect plots for drivers of flood mortality, excluding observations for India. Each plot (a-n) shows the in-sample median of the posterior predictive distribution (black line) and the surrounding 80% predictive interval (shaded area) for the selected indicator, based on 8 × 4,000 Markov chain Monte Carlo draws per model. All models are specified as Bayesian random-effects negative binomial regressions. All effects are computed by including the selected indicator of political development only (color), in addition to the baseline predictors (grey), holding other variables at their observed mean values. Colors reflect the political development dimension of the main predictor in the model (blue: democracy, pink: institutional quality, orange: peace breakdown). Rug plots display the distribution of data points. Plots are shown for a subset of observations excluding the 20% most severe conflict history events. The underlying models were estimated on a restricted training sample which excludes observations for India, 2000–14 (N=1,794).

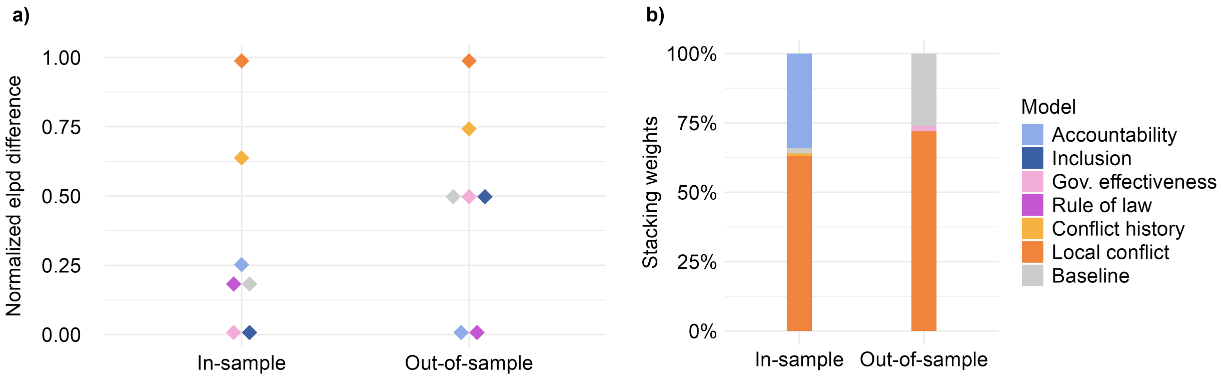

c)

	In-sample (2000-2014)			Out-of-sample (2015-2018)		
Model	elpd LOO	Δelpd	stacking weight	elpd LOO	Δelpd	stacking weight
Local conflict	-4295 (110)	0 (0)	0.63	-480 (34)	0 (0)	0.72
Conflict history	-4301 (110)	-6 (6)	0.01	-481 (34)	-1 (1)	0.00
Accountability	-4308 (110)	-13 (10)	0.34	-483 (34)	-4 (2)	0.00
Rule of law	-4309 (110)	-14 (8)	0.00	-483 (34)	-4 (2)	0.00
Baseline	-4310 (111)	-14 (8)	0.02	-482 (34)	-2 (2)	0.26
Gov. effectiveness	-4312 (111)	-17 (8)	0.00	-482 (34)	-2 (2)	0.02
Inclusion	-4313 (111)	-17 (9)	0.00	-482 (34)	-2 (2)	0.00

Fig. S33. Relative predictive performance of political development models on flood mortality, excluding observations for India. a) In-sample (2000–14, N=1,794) and out-of-sample (2015–18, N=286) expected log predictive density (elpd) for each model, obtained via leave-one-out cross-validation with Pareto-smoothed importance sampling, normalized such that the best-performing model scores 1 and the worst scores 0. **b)** In-sample and out-of-sample stacking weights for each model, reflecting the proportion of observations for which each model provides superior predictive performance. **c)** In-sample and out-of-sample elpd values (non-normalized) and stacking weights for each indicator model, ranked by in-sample elpd performance (values closer to zero indicate better fit; standard errors in parentheses). Each model includes all indicators of a given political development dimension (color) in addition to the baseline (grey). Colors reflect the political development dimension of the main predictor in the model (blue: democracy, pink: institutional quality, orange: peace breakdown).

2.10.4 Excluding China

Another frequently flood-exposed country is China, with 135 events recorded for the 2000–18 period (avg. mortality per event=34.47). The following figures show results without China.

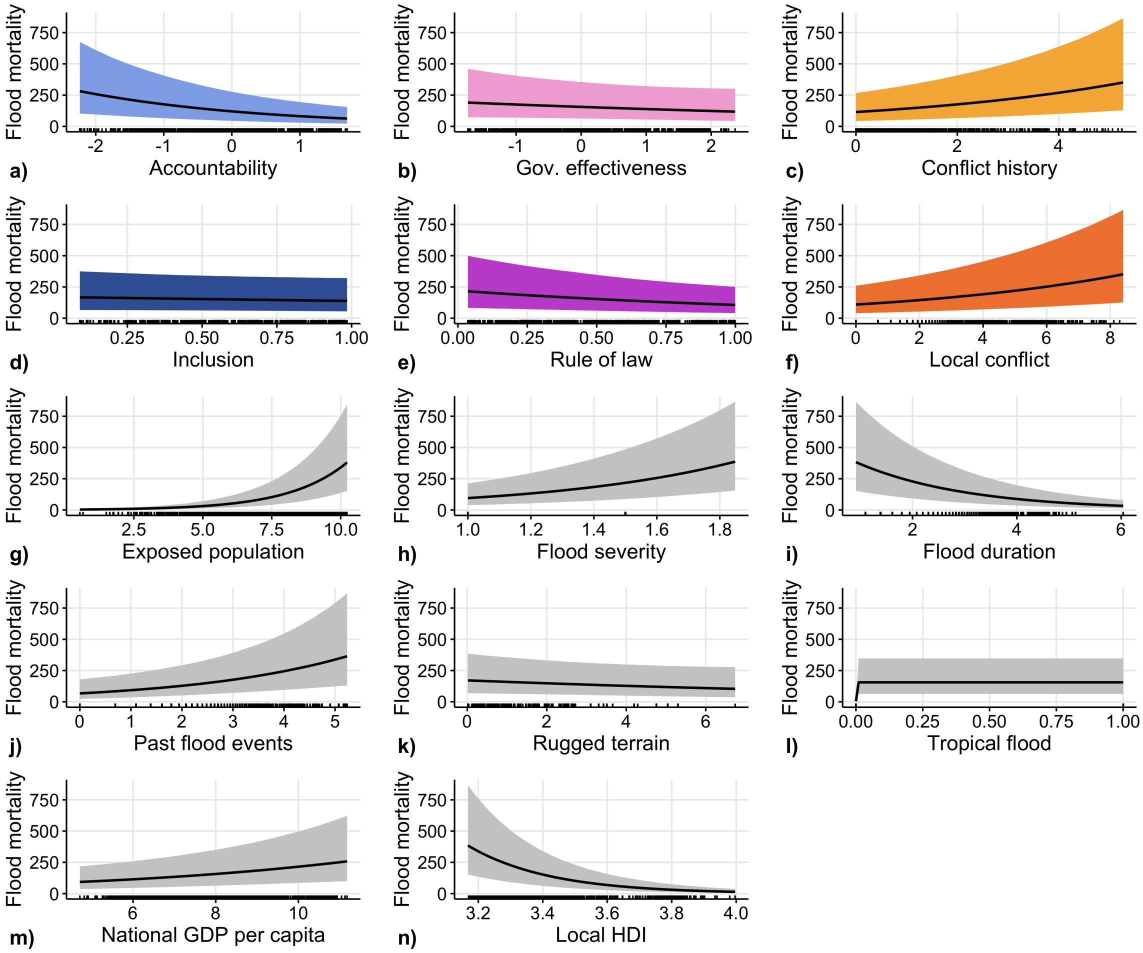

Fig. S34. Conditional effect plots for all drivers of flood mortality, excluding observations for China. Each plot (a-n) shows the in-sample median of the posterior predictive distribution (black line) and the surrounding 80% predictive interval (shaded area) for the selected indicator, based on 8 × 4,000 Markov chain Monte Carlo draws per model. All models are specified as Bayesian random-effects negative binomial regressions. All effects are computed by including the selected indicator of political development only (color), in addition to the baseline predictors (grey), holding other variables at their observed mean values. Colors reflect the political development dimension of the main predictor in the model (blue: democracy, pink: institutional quality, orange: peace breakdown). Rug plots display the distribution of data points. Plots are shown for a subset of observations excluding the 20% most severe conflict history events, although the underlying models were estimated on a restricted training sample that excludes observations for China, 2000–14 (N=1,796).

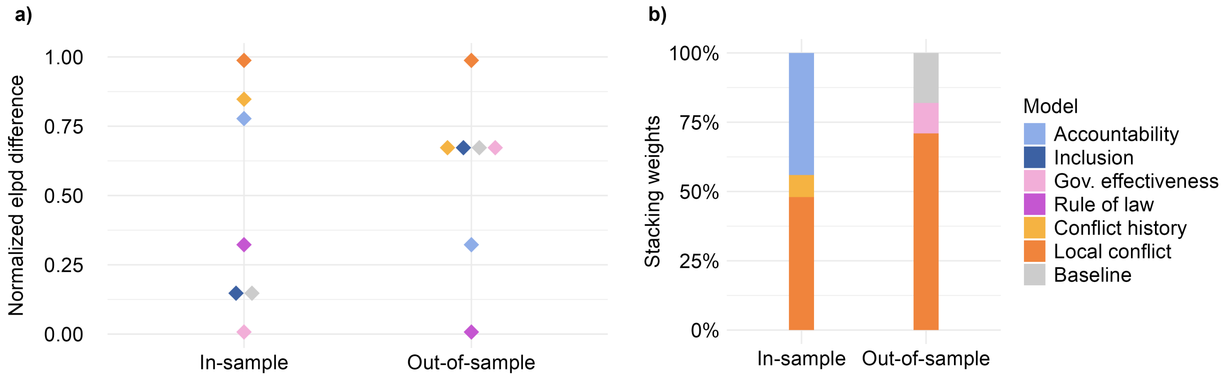

c)

Model	In-sample (2000-2014)			Out-of-sample (2015-2018)		
	elpd LOO	Δelpd	stacking weight	elpd LOO	Δelpd	stacking weight
Local conflict	-4596 (114)	0 (0)	0.48	-569 (37)	0 (0)	0.71
Conflict history	-4598 (113)	-2 (6)	0.08	-570 (37)	-1 (1)	0.00
Accountability	-4599 (113)	-3 (9)	0.44	-572 (37)	-2 (2)	0.00
Rule of law	-4605 (114)	-9 (7)	0.00	-572 (37)	-3 (1)	0.00
Inclusion	-4606 (114)	-11 (7)	0.00	-571 (37)	-1 (1)	0.00
Baseline	-4607 (114)	-11 (7)	0.00	-570 (37)	-1 (1)	0.18
Gov. effectiveness	-4609 (114)	-13 (7)	0.00	-570 (37)	-1 (1)	0.11

Fig. S35. Relative predictive performance of political development models on flood mortality, excluding observations for China. a) In-sample (2000–14, N=1,796) and out-of-sample (2015–18, N=294) expected log predictive density (elpd) for each model, obtained via leave-one-out cross-validation with Pareto-smoothed importance sampling, normalized such that the best-performing model scores 1 and the worst scores 0. **b)** In-sample and out-of-sample stacking weights for each model, reflecting the proportion of observations for which each model provides superior predictive performance. **c)** In-sample and out-of-sample elpd values (non-normalized) and stacking weights for each indicator model, ranked by in-sample elpd performance (values closer to zero indicate better fit; standard errors in parentheses). Each model includes all indicators of a given political development dimension (color) in addition to the baseline (grey). Colors reflect the political development dimension of the main predictor in the model (blue: democracy, pink: institutional quality, orange: peace breakdown).

2.10.5 Excluding Bangladesh

Bangladesh also experienced many flood events in our dataset (N= 101, avg. mortality per event=60.83). The following figures show results without Bangladesh.

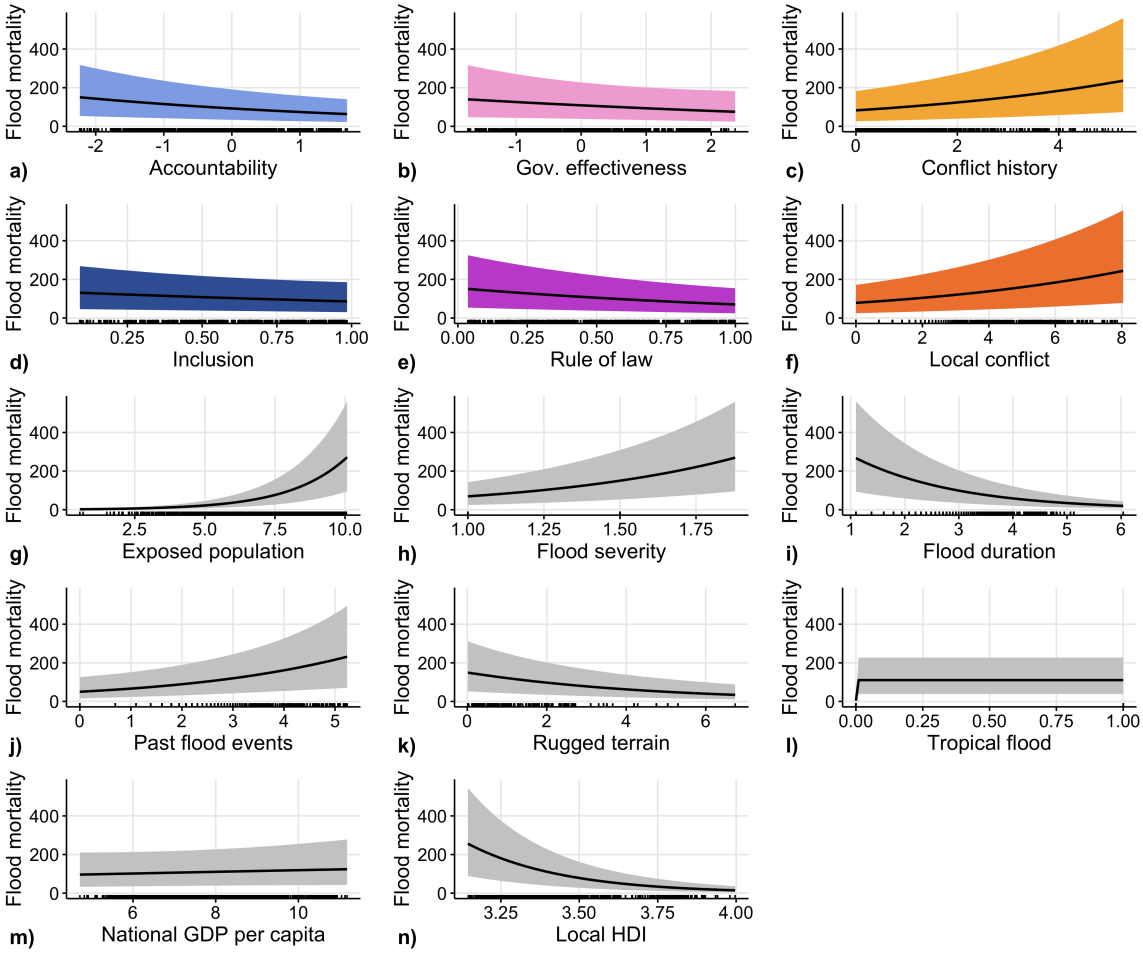

Fig. S36. Conditional effect plots for all drivers of flood mortality, excluding observations for Bangladesh. Each plot (a-n) shows the in-sample median of the posterior predictive distribution (black line) and the surrounding 80% predictive interval (shaded area) for the selected indicator, based on 8 × 4,000 Markov chain Monte Carlo draws per model. All models are specified as Bayesian random-effects negative binomial regressions. All effects are computed by including the selected indicator of political development only (color), in addition to the baseline predictors (grey), holding other variables at their observed mean values. Colors reflect the political development dimension of the main predictor in the model (blue: democracy, pink: institutional quality, orange: peace breakdown). Rug plots display the distribution of data points. Plots are shown for a subset of observations excluding the 20% most severe conflict history events, although the underlying models were estimated on a restricted training sample that excludes observations for Bangladesh, 2000–14 (N=1,830).

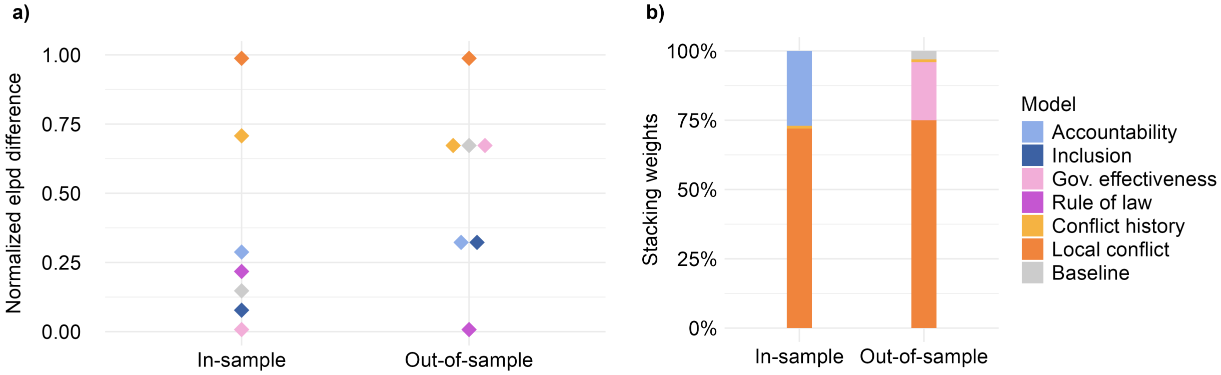

c)

	In-sample (2000-2014)			Out-of-sample (2015-2018)		
Model	elpd LOO	Δelpd	stacking weight	elpd LOO	Δelpd	stacking weight
Local conflict	-4632 (115)	0 (0)	0.72	-544 (36)	0 (0)	0.75
Conflict history	-4636 (114)	-4 (5)	0.01	-545 (37)	-1 (1)	0.01
Accountability	-4642 (115)	-10 (8)	0.27	-546 (37)	-2 (1)	0.00
Rule of law	-4643 (115)	-11 (7)	0.00	-547 (37)	-3 (1)	0.00
Baseline	-4644 (116)	-12 (7)	0.00	-545 (36)	-1 (1)	0.03
Inclusion	-4645 (115)	-13 (7)	0.00	-546 (37)	-2 (1)	0.00
Gov. effectiveness	-4645 (116)	-14 (7)	0.00	-545 (37)	-1 (1)	0.21

Fig. S37. Relative predictive performance of political development models on flood mortality, excluding observations for Bangladesh. a) In-sample (2000–14, N=1,830) and out-of-sample (2015–18, N=294) expected log predictive density (elpd) for each model, obtained via leave-one-out cross-validation with Pareto-smoothed importance sampling, normalized such that the best-performing model scores 1 and the worst scores 0. **b)** In-sample and out-of-sample stacking weights for each model, reflecting the proportion of observations for which each model provides superior predictive performance. **c)** In-sample and out-of-sample elpd values (non-normalized) and stacking weights for each indicator model, ranked by in-sample elpd performance (values closer to zero indicate better fit; standard errors in parentheses). Each model includes all indicators of a given political development dimension (color) in addition to the baseline (grey). Colors reflect the political development dimension of the main predictor in the model (blue: democracy, pink: institutional quality, orange: peace breakdown).

2.11 Alternative estimation strategy: random forest models

Figure S38 presents the Global SHAP values for all political development indicators, measuring their average contribution to the predictions. SHAP values are computed by training a set of Random Forest machine learning models where each political development predictor is individually added to the baseline indicators, similarly to the main specification presented in the article. Random forest is a machine learning algorithm that builds an ensemble of decision trees and combines their predictions to improve accuracy and reduce over-fitting – making it a robust and flexible approach to predicting [5].

SHAP (SHapley Additive exPlanations) values are a measure of the contribution of each predictor to a specific prediction instance. Global SHAP values, presented in the Figure, are obtained by averaging the individual SHAP values across all predictions for the test set. Global SHAP values thus represent a measure of the average contribution of each indicator to the overall predictions. The SHAP approach is based on coalitional game theory, where features are treated like players in a coalition. Shapley values are computed by considering all possible combinations (coalitions) of predictors being either included or excluded from the 'prediction game'. The SHAP approach represents a valuable method for interpreting the results of machine learning models, while maintaining key properties of fairness and consistency in feature importance attribution, and they are similar to permutation importance scores [6].

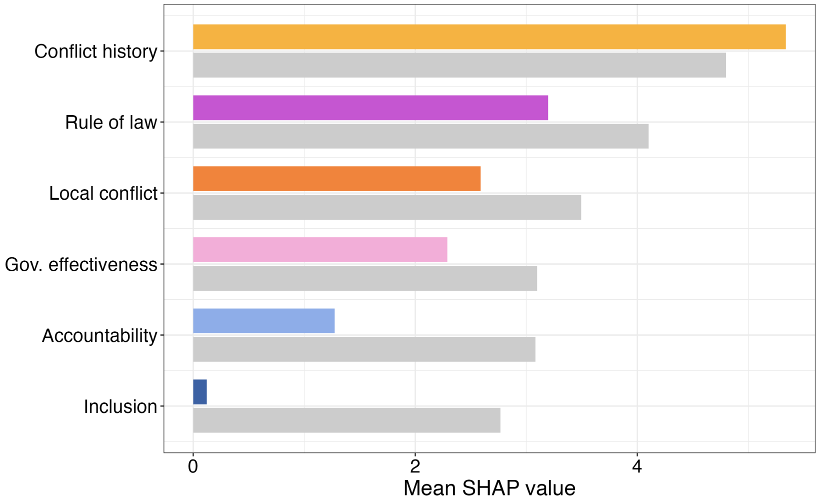

Fig. S38. Global SHAP values for all political development indicators (color) relative to the baseline (grey). Models are trained on the training set for 2000-2014 (N=1,914) using a Random Forest algorithm with 500 trees, and adding each political development indicator to the baseline as in the main specification. Colors reflect the political development dimension of the main predictor in the model (blue: democracy, pink: institutional quality, orange: peace breakdown). Global SHAP values are computed as the mean absolute Shapley values across all observations in the test set; values for the baseline are presented as average across all baseline indicators for a given model.

2.12 Alternative estimation strategy: fixed effects models

Table S6 and Figure S39 present the effect of political development on flood mortality estimated using Poisson regression models with two-way fixed effects (country and year). In Figure S39, each point on the plot represents the incidence rate ratio for a given predictor. The error bars around the points indicate 95% confidence intervals. The results are in line with those presented in the main article.

	Models						
	**Baseline**	**Accountability**	**Inclusion**	**Gov. effectiveness**	**Rule of law**	**Conflict history**	**Local conflict**
Exposed population	1.774***	1.822***	1.773***	1.762***	1.766***	1.737***	1.612***
	[1.398, 2.252]	[1.436, 2.311]	[1.400, 2.246]	[1.405, 2.210]	[1.394, 2.238]	[1.398, 2.158]	[1.292, 2.012]
	(0.000)	(0.000)	(0.000)	(0.000)	(0.000)	(0.000)	(0.000)
Flood severity	0.946	1.044	0.954	1.057	0.956	1.016	0.970
	[0.475, 1.885]	[0.529, 2.059]	[0.484, 1.880]	[0.510, 2.191]	[0.483, 1.894]	[0.501, 2.063]	[0.564, 1.667]
	(0.876)	(0.902)	(0.892)	(0.881)	(0.898)	(0.964)	(0.911)
Flood duration	1.458***	1.408**	1.458***	1.436**	1.456**	1.411**	1.271**
	[1.098, 1.936]	[1.069, 1.853]	[1.096, 1.939]	[1.078, 1.913]	[1.092, 1.942]	[1.073, 1.854]	[1.013, 1.594]
	(0.009)	(0.015)	(0.010)	(0.013)	(0.011)	(0.014)	(0.038)
Past flood events	6.807**	8.178**	7.036**	4.377**	7.517***	4.375**	6.354**
	[1.470, 31.509]	[1.619, 41.316]	[1.492, 33.181]	[1.210, 15.838]	[1.784, 31.664]	[1.317, 14.536]	[1.343, 30.055]
	(0.014)	(0.011)	(0.014)	(0.024)	(0.006)	(0.016)	(0.020)
National GDP per capita	2.048	2.898	2.310	4.499**	2.754	3.952**	3.625
	[0.398, 10.521]	[0.543, 15.473]	[0.394, 13.538]	[1.062, 19.053]	[0.582, 13.039]	[1.132, 13.792]	[0.634, 20.726]
	(0.391)	(0.213)	(0.353)	(0.041)	(0.202)	(0.031)	(0.148)
Local HDI	0.357	0.286	0.336	0.178	0.338	0.876	0.175
	[0.005, 26.867]	[0.004, 22.569]	[0.004, 26.142]	[0.002, 13.855]	[0.004, 28.793]	[0.009, 82.864]	[0.003, 11.892]
	(0.640)	(0.575)	(0.623)	(0.437)	(0.632)	(0.955)	(0.418)
Accountability		0.148***					
		[0.042, 0.527]					
		(0.003)					
Inclusion			0.036				
			[0.000, 16.871]				
			(0.290)				
Gov. effectiveness				0.110**			
				[0.016, 0.740]			
				(0.023)			
Rule of law					0.012		
					[0.000, 3.249]		
					(0.121)		
Conflict history						2.041***	
						[1.249, 3.334]	
						(0.004)	
Local conflict							1.487***
							[1.242, 1.779]
							(0.000)
Num.Obs.	2108	2108	2108	2108	2108	2108	2108
RMSE	253.62	255.09	252.92	244.66	251.34	236.51	208.84
Country fixed effects	X	X	X	X	X	X	X
Year fixed effects	X	X	X	X	X	X	X

Table S6. Poisson fixed effects models. Incidence Rate Ratios. 95% confidence intervals are displayed in brackets, exact p-values in parentheses. The coefficients are obtained from two-way fixed effect Poisson regression models with country and year fixed effects, adding each political development indicator to the baseline as in the main specification (each political development indicator is tested in a separate model). No adjustments were made for multiple comparisons. † $p < 0.1$, ** $p < 0.05$, *** $p < 0.01$

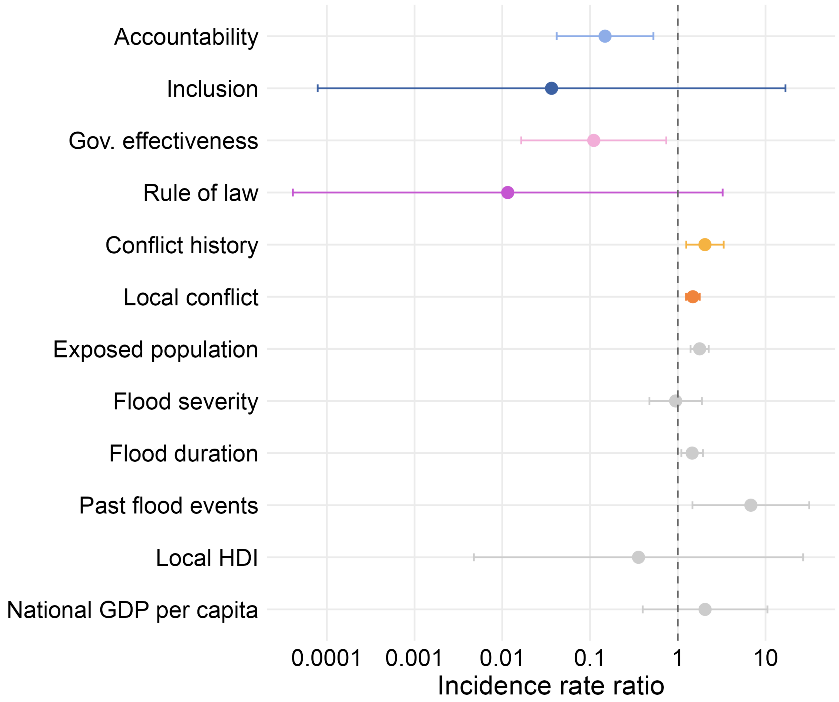

Fig. S39. Effect of political development on flood mortality. The coefficients are obtained from two-way fixed effect Poisson regression models with country and year fixed effects, adding each political development indicator to the baseline as in the main specification (each political development indicator is tested in a separate model). Colors reflect the political development dimension of the main predictor in the model (blue: democracy, pink: institutional quality, orange: peace breakdown). No adjustments were made for multiple comparisons. Corresponding p-values for each coefficient are reported in Table S6.

3 Counterfactual analysis

This section details the methods underlying the counterfactual analysis presented in Figure 5, and discusses its main limitations and caveats. The counterfactual analysis compares predictions obtained for the observed data against predictions obtained for a simulated dataset where the values of all political development indicators, across all countries and years in the test set, are set identical to the observed values for New Zealand (Fig. 5) or Ghana (Fig. S40) as recorded in 2018 in our dataset.[1] New Zealand scores in the top 10% or higher across all political indicators (with the exception of Inclusion which is only slightly lower than 10% – cf. Table S7); the country hence serves as an example of an ideal case where SDG 16 has been achieved. Ghana in 2018 is chosen as an example of a low-income country with moderate political development and no conflict, which may be considered a more realistic target for non-democratic and conflict-affected low-income countries in the near term (Table S7). Although Ghana did not experience recent conflict, the other indicators of political development are considerably lower than the top 10th percentile (but relatively high considering the national GDP per capita).

Table S7. Political development scores for New Zealand and Ghana (2018) vs. full dataset, 2015–18.*

Political indicator	New Zealand	Ghana	Full test set		
			Top 10%	Top 5%	Top 1%
Accountability	1.56	0.60	1.02	1.14	1.37
Inclusion	0.94	0.81	0.95	0.96	0.97
Gov. effectiveness	1.76	-0.16	1.21	1.55	1.85
Rule of law	0.99	0.60	0.96	0.98	0.99
Conflict history	0	0	0	0	0
Local conflict	0	0	0	0	0
GDP per capita	42,911	1,999	23,514	55,124	59,908
Local HDI	42.56	23.12	38.25	45.53	50.42

**Note:* The table details the values of political development indicators and GDP per capita for New Zealand and Ghana (2018), relative to the distribution of observed values in the test set, 2015–18 (N=311). 90th, 95th, and 99th percentiles are reported for indicators where higher scores indicate positive developments (e.g. Accountability); 10th, 5th, and 1st percentile are used for indicators where lower scores indicate positive development (e.g. Conflict). Values for GDP per capita and Local HDI (unlogged) are reported for comparison, although they remain unchanged in the counterfactual analysis. Note that since all predictors are lagged by 1 year in our data structure, in the counterfactual exercise, we use their actual value in 2017 (which correspond to 2018 in our data structure).

The counterfactual analysis was performed as follows. First, we generated posterior distributions for actual data and for the counterfactual scenario, using the same sets of models, for the test set. Second, we computed the average posterior distribution for each flood event across all 32,000 samples, for both the actual data and the counterfactual.

[1]Note that since all predictors are lagged by one year in our data structure, the 2018 values used in the counterfactual correspond to New Zealand's actual political conditions in 2017. The same applies to the counterfactual for Ghana. This ensures consistency with how predictor variables are structured throughout our analysis.

Third, we computed the aggregate mean across all flood events (and across samples) for the actual data and the New Zealand (or Ghana) scenario.

A few important caveats apply when interpreting the results of such counterfactual analysis. First, the posterior distribution is based on models, and thus suffers from the same limitation as the models themselves. Crucially, our dataset on flood mortality and political development is characterized by strong temporal and spatial dependencies: if our model is unable to fully account for these dependencies, the predictions – and therefore the results of the counterfactual analysis – might be inaccurate. Second, a counterfactual analysis represents by definition an imaginary scenario. In this context, we create data of fictitious countries that were exposed to the same flood events and featured the same baseline characteristics as in the actual world, but exhibited the level of political development as New Zealand (or Ghana) in 2018. While the analysis can be used for illustrating the implications of a hypothetical state of the world with high political development, it may not represent a likely development trajectory. Finally, the aggregate predictions might suffer from downward or upward bias, especially if the models are better at predicting certain types of flood events or patterns in specific regions or times – which may lead to over- or under-estimation in the aggregated predictions presented in Figure 5.

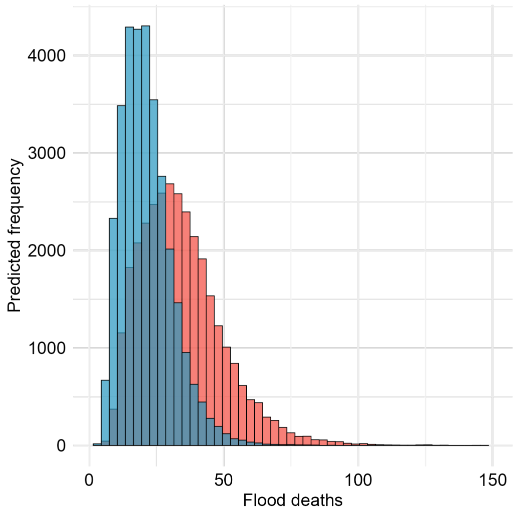

a)

Model	$\hat{y}_{obs}$	$\hat{y}_{cnt}$	$\Delta\hat{y}$	$\Delta\hat{y}$ (range)
Accountability	102.92	89.58	−13%	0 – 171.53
Inclusion	116.40	113.36	−3%	0 – 167.84
Gov. effectiveness	113.81	117.78	3%	0 – -67.77
Rule of law	113.88	110.72	−3%	0 – -362.11
Conflict history	90.40	76.98	−15%	0 – 614.08
Local conflict	114.07	70.35	−38%	0 – 5,474.46
All pol. predictors	92.57	46.69	−50%	0.01 – 3,058.87

b)

Fig. S40. Counterfactual out-of-sample analysis of flood mortality. (a) 90th percentile posterior predictive distribution of flood mortality across all flood events, 2015–18 (N=311), obtained from models using observed (red) or counterfactual (blue) values on all political predictors. (b) Observed and counterfactual out-of-sample predictions for individual political predictor models and the model containing all predictors. $\hat{y}_{obs}$ represents the average of the mean prediction using observed predictor values; $\hat{y}_{cnt}$ gives corresponding average predictions based on counterfactual predictor values set identical to Ghana in 2018. $\Delta\hat{y}$ gives the relative difference in ensemble average predictions between observed and counterfactual models. The final column reports the range of difference in the average predicted flood mortality across all 32,000 samples.

References

[1] Kaufmann, D. & Kraay, A. Worldwide governance indicators, 2023 update (2023). Available at: https://info.worldbank.org/governance/wgi/.

[2] Davies, S., Pettersson, T. & Öberg, M. Organized violence 1989–2021 and drone warfare. *Journal of Peace Research* **59**, 593–610 (2022).

[3] Smits, J. & Permanyer, I. The Subnational Human Development Database. *Scientific Data* **6**, 190038 (2019).

[4] Coppedge, M. *et al.* V-dem country-year dataset v13 (2023). URL https://doi.org/10.23696/vdemds21. Available at: https://v-dem.net.

[5] Breiman, L. Random forests. *Machine learning* **45**, 5–32 (2001).

[6] Molnar, C. *Interpretable Machine Learning: A Guide for Making Black Box Models Explainable* (2022). URL https://christophm.github.io/interpretable-ml-book/. Accessed: 2024-09-26.
