# Supplementary material for: Political development predicts reduced human cost of flooding
Source: Nat Commun. 2025 Nov 26;16:10128. doi: 10.1038/s41467-025-65914-6 (PMC12657876; doi:10.1038/s41467-025-65914-6)
Supplement: Supplementary file 2 — Reporting Summary [file 41467_2025_65914_MOESM2_ESM.pdf]

nature portfolio

Corresponding author(s): Paola Vesco

Last updated by author(s): Oct 13, 2025

Reporting Summary

Nature Portfolio wishes to improve the reproducibility of the work that we publish. This form provides structure for consistency and transparency in reporting. For further information on Nature Portfolio policies, see our Editorial Policies and the Editorial Policy Checklist.

Statistics

For all statistical analyses, confirm that the following items are present in the figure legend, table legend, main text, or Methods section.

n/a	Confirmed	
☐	☒	The exact sample size (*n*) for each experimental group/condition, given as a discrete number and unit of measurement
☒	☐	A statement on whether measurements were taken from distinct samples or whether the same sample was measured repeatedly
☐	☒	The statistical test(s) used AND whether they are one- or two-sided<br>*Only common tests should be described solely by name; describe more complex techniques in the Methods section.*
☐	☒	A description of all covariates tested
☐	☒	A description of any assumptions or corrections, such as tests of normality and adjustment for multiple comparisons
☐	☒	A full description of the statistical parameters including central tendency (e.g. means) or other basic estimates (e.g. regression coefficient) AND variation (e.g. standard deviation) or associated estimates of uncertainty (e.g. confidence intervals)
☐	☒	For null hypothesis testing, the test statistic (e.g. *F*, *t*, *r*) with confidence intervals, effect sizes, degrees of freedom and *P* value noted<br>*Give P values as exact values whenever suitable.*
☐	☒	For Bayesian analysis, information on the choice of priors and Markov chain Monte Carlo settings
☐	☒	For hierarchical and complex designs, identification of the appropriate level for tests and full reporting of outcomes
☐	☒	Estimates of effect sizes (e.g. Cohen's *d*, Pearson's *r*), indicating how they were calculated

Our web collection on statistics for biologists contains articles on many of the points above.

Software and code

Policy information about availability of computer code

Data collection	R version 4.3.2.
Data analysis	R version 4.3.2, brms v2.22.1. Code to reproduce the analysis is available at https://github.com/prio-data/political_development_flood and archived at https://doi.org/10.5281/zenodo.17314421117.

For manuscripts utilizing custom algorithms or software that are central to the research but not yet described in published literature, software must be made available to editors and reviewers. We strongly encourage code deposition in a community repository (e.g. GitHub). See the Nature Portfolio guidelines for submitting code & software for further information.

Data

Policy information about availability of data

All manuscripts must include a data availability statement. This statement should provide the following information, where applicable:

- Accession codes, unique identifiers, or web links for publicly available datasets
- A description of any restrictions on data availability
- For clinical datasets or third party data, please ensure that the statement adheres to our policy

All source data are freely available, and sources are indicated in the Methods section. Data on floods worldwide, including flood severity, duration, tropical storm-related floods, and count of past flood events, are provided by the Global Flood Database and available from: https://global-flood-database.cloudtostreet.ai/. Gridded data on population exposed to flooding were prepared by Jonas Vestby and co-authors for their article "Societal determinants of flood-induced

displacement" published in PNAS 121 (3), 2024, and can be downloaded from: https://dataverse.harvard.edu/dataset.xhtml?persistentId=doi:10.7910/DVN/JMAP2M. Dartmouth Flood Observatory (DFO) data on flood mortality are available from: https://floodobservatory.colorado.edu/Archives/. Country-year data on governmental accountability and effectiveness are provided by the Worldwide Governance Indicators (v2023) and are available from: https://www.worldbank.org/en/publication/worldwide-governance-indicators. Country-year indicators of inclusion and rule of law are provided by V-Dem (v13) and can be downloaded from: https://www.v-dem.net/data/the-v-dem-dataset/. Geo-referenced data on armed conflict (v.21.1) are available from the Uppsala Conflict Data Program: https://ucdp.uu.se/downloads/. Country-level data on terrain ruggedness are provided by Nathan Nunn and Diego Puga for their article "Ruggedness: The blessing of bad geography in Africa" published in the Review of Economics and Statistics 94(1), 2012, and can be downloaded from: https://diegopuga.org/data/rugged/. Country-year statistics on Gross Domestic Product are available from the World Bank's World Development Indicators at: https://databank.worldbank.org/source/world-development-indicators. Sub-national level data on local HDI are provided by sub-HDI and can be downloaded from: https://globaldatalab.org/shdi/. CShapes GIS data of country boundaries are available from https://cran.r-project.org/web/packages/cshapes/index.html. Replication data to reproduce the results reported here, which were created from these sources, have been deposited in Harvard Dataverse with accession code https://doi.org/10.7910/DVN/YMUDX8 .

Research involving human participants, their data, or biological material

Policy information about studies with human participants or human data. See also policy information about sex, gender (identity/presentation), and sexual orientation and race, ethnicity and racism.

Reporting on sex and gender	No data involving human participants or individual level data are used in this study. Aggregate population characteristics are presented in the Methods section of the manuscript and in the Supplementary Information.
Reporting on race, ethnicity, or other socially relevant groupings	No data involving human participants or individual level data are used in this study. Aggregate population characteristics are presented in the Methods section of the manuscript and in the Supplementary Information.
Population characteristics	No data involving human participants or individual level data are used in this study. Aggregate population characteristics are presented in the Methods section of the manuscript and in the Supplementary Information.
Recruitment	No data involving human participants or individual level data are used in this study.
Ethics oversight	N/A

Note that full information on the approval of the study protocol must also be provided in the manuscript.

Field-specific reporting

Please select the one below that is the best fit for your research. If you are not sure, read the appropriate sections before making your selection.

☐ Life sciences ☒ Behavioural & social sciences ☐ Ecological, evolutionary & environmental sciences

For a reference copy of the document with all sections, see nature.com/documents/nr-reporting-summary-flat.pdf

Behavioural & social sciences study design

All studies must disclose on these points even when the disclosure is negative.

Study description	Quantitative design, using a combination of Bayesian negative binomial regression and in-sample and out-of-sample validation of the models' predictive performance.
Research sample	N = 2,225
Sampling strategy	N/A
Data collection	No data were collected for this study. All data are drawn from open access sources.
Timing	N/A
Data exclusions	Flood events occurring in uninhabited areas where excluded from the sample.
Non-participation	N/A
Randomization	N/A

Reporting for specific materials, systems and methods

We require information from authors about some types of materials, experimental systems and methods used in many studies. Here, indicate whether each material, system or method listed is relevant to your study. If you are not sure if a list item applies to your research, read the appropriate section before selecting a response.

Materials & experimental systems

n/a	Involved in the study
☒	☐ Antibodies
☒	☐ Eukaryotic cell lines
☒	☐ Palaeontology and archaeology
☒	☐ Animals and other organisms
☒	☐ Clinical data
☒	☐ Dual use research of concern
☒	☐ Plants

Methods

n/a	Involved in the study
☒	☐ ChIP-seq
☒	☐ Flow cytometry
☒	☐ MRI-based neuroimaging

Plants

Seed stocks	N/A
Novel plant genotypes	N/A
Authentication	N/A
